# Genetic Polymorphism at *CCL5* Is Associated With Protection in Chagas’ Heart Disease: Antagonistic Participation of CCR1^+^ and CCR5^+^ Cells in Chronic Chagasic Cardiomyopathy

**DOI:** 10.3389/fimmu.2018.00615

**Published:** 2018-04-11

**Authors:** Angelica Martins Batista, Lucia Elena Alvarado-Arnez, Silvia Marinho Alves, Gloria Melo, Isabela Resende Pereira, Leonardo Alexandre de Souza Ruivo, Andrea Alice da Silva, Daniel Gibaldi, Thayse do E. S. Protásio da Silva, Virginia Maria Barros de Lorena, Adriene Siqueira de Melo, Ana Karine de Araújo Soares, Michelle da Silva Barros, Vláudia Maria Assis Costa, Cynthia C. Cardoso, Antonio G. Pacheco, Cristina Carrazzone, Wilson Oliveira, Milton Ozório Moraes, Joseli Lannes-Vieira

**Affiliations:** ^1^Laboratório de Biologia das Interações, Instituto Oswaldo Cruz, Fundação Oswaldo Cruz (Fiocruz), Rio de Janeiro, Brazil; ^2^Laboratório de Hanseníase, Instituto Oswaldo Cruz, Fundação Oswaldo Cruz (Fiocruz), Rio de Janeiro, Brazil; ^3^Ambulatório de Doença de Chagas e Insuficiência Cardíaca do Pronto Socorro Cardiológico de Pernambuco (PROCAPE/UPE), Recife, Brazil; ^4^Laboratório Multiusuário de Apoio à Pesquisa em Nefrologia e Ciências Médicas, Departamento de Patologia, Faculdade de Medicina, Universidade Federal Fluminense, Rio de Janeiro, Brazil; ^5^Laboratório de Imunoparasitologia, Departamento de Imunologia, Instituto Aggeu Magalhães, Fundação Oswaldo Cruz (Fiocruz), Recife, Brazil; ^6^Departamento de Medicina Tropical, Universidade Federal de Pernambuco, Recife, Brazil; ^7^Laboratório de Imunopatologia Keizo Asami (LIKA), Universidade Federal de Pernambuco, Recife, Brazil; ^8^Laboratório de Virologia Molecular, Departamento de Genética, Universidade Federal do Rio de Janeiro, Rio de Janeiro, Brazil; ^9^Programa de Computação Científica, Fundação Oswaldo Cruz (Fiocruz), Rio de Janeiro, Brazil

**Keywords:** Chagas disease, *Trypanosoma cruzi*, heart disease, cell migration, CCL5, CCR1, CCR5, Met-RANTES

## Abstract

Chronic cardiomyopathy is the main clinical manifestation of Chagas disease (CD), a disease caused by *Trypanosoma cruzi* infection. A hallmark of chronic chagasic cardiomyopathy (CCC) is a fibrogenic inflammation mainly composed of CD8^+^ and CD4^+^ T cells and macrophages. CC-chemokine ligands and receptors have been proposed to drive cell migration toward the heart tissue of CD patients. Single nucleotide polymorphisms (SNPs) in CC-chemokine ligand and receptor genes may determine protein expression. Herein, we evaluated the association of SNPs in the CC-chemokines *CCL2* (rs1024611) and *CCL5* (rs2107538, rs2280788) and the CCL5/RANTES receptors *CCR1* (rs3181077, rs1491961, rs3136672) and *CCR5* (rs1799987) with risk and progression toward CCC. We performed a cross-sectional association study of 406 seropositive patients from endemic areas for CD in the State of Pernambuco, Northeast Brazil. The patients were classified as non-cardiopathic (A, *n* = 110) or cardiopathic (mild, B1, *n* = 163; severe, C, *n* = 133). Serum levels of CCL5 and CCL2/MCP-1 were elevated in CD patients but were neither associated with risk/severity of CCC nor with SNP genotypes. After logistic regression analysis with adjustment for the covariates gender and ethnicity, *CCL5* −403 (rs2107538) CT heterozygotes (OR = 0.5, *P*-value = 0.04) and T carriers (OR = 0.5, *P*-value = 0.01) were associated with protection against CCC. To gain insight into the participation of the CCL5–CCR5/CCR1 axis in CCC, mice were infected with the Colombian *T. cruzi* strain. Increased CCL5 concentrations were detected in cardiac tissue. In spleen, frequencies of CCR1^+^ CD8^+^ T cells and CD14^+^ macrophages were decreased, while frequencies of CCR5^+^ cells were increased. Importantly, CCR1^+^CD14^+^ macrophages were mainly IL-10^+^, while CCR5^+^ cells were mostly TNF^+^. CCR5-deficient infected mice presented reduced TNF concentrations and injury in heart tissue. Selective blockade of CCR1 (Met-RANTES therapy) in infected *Ccr5*^−/−^ mice supported a protective role for CCR1 in CCC. Furthermore, parasite antigen stimulation of CD patient blood cells increased the frequency of CCR1^+^CD8^+^ T cells and CCL5 production. Collectively, our data support that a genetic variant of *CCL5* and CCR1^+^ cells confer protection against Chagas heart disease, identifying the CCL5-CCR1 axis as a target for immunostimulation.

## Introduction

Chagas disease (CD) is a neglected tropical disease caused by the protozoan parasite *Trypanosoma cruzi*. CD affects 6 to 7 million people worldwide, representing a major public health concern, mainly due to vector transmission in Latin America (1). Due to human migration, 1 to 2 million CD patients are living in the USA, Europe, and Asia, where parasite transmission poses a risk through organ transplantation, blood transfusion, and pregnancy (2). Decades after the initial infection, 60–70% of CD patients remain asymptomatic with no clinical manifestations, whereas 20–40% of patients develop the cardiac form. Chronic chagasic cardiomyopathy (CCC), a life-threatening form of inflammatory dilated cardiomyopathy, is the most frequent expression of CD with symptoms that range from mild to severe and widespread cardiac remodeling associated with fibrosis, arrhythmias, and thromboembolic events, which may culminate in congestive heart failure (CHF) and sudden death (2, 3). The intensity of fibrogenic heart inflammation, mainly composed of CD8^+^ and CD4^+^ T-cells and macrophages, is linked to CCC severity (4, 5). In accordance with this differential accumulation of mononuclear cells, CC-chemokines and their receptors have been proposed to play a crucial role in the recruitment and migration of circulating cells toward the heart tissue in chronic CD, both in humans and experimental models (6). Importantly, in non-chagasic heart diseases, innate and adaptive immune cells, particularly spleen-born T cells and macrophages, are involved in both heart tissue injury and repair (7, 8).

Chemokines are small (8–14 kDa) proteins that constitute the largest subfamily of cytokines. Their biological activity is mediated by specific G protein-coupled receptors expressed on the surface of several cell types (9–11). CC-chemokine ligands and receptors are involved in the pathogenesis of cardiac and infectious diseases (12, 13). In chronic CD, CCL5/RANTES and CCL2/MCP-1 serum concentrations and the frequencies of peripheral blood CCR5^+^ T cells and macrophages are increased in cardiopathic patients (14, 15). The beneficial effects of Met-RANTES, a partial antagonist of CCR1/CCR5-mediated interactions, have supported the participation of CC-chemokine ligands and receptors in cell migration, myocarditis formation, and heart tissue damage in experimental models of CCC (16, 17). Nevertheless, the CC-chemokines CCL5 and CCL2 trigger effector functions of macrophages and participate in parasite growth control (18, 19).

The expression of chemokine ligands and receptors is partially controlled by genetic polymorphisms, potentially contributing to differential patterns of cell migration and effector activities in health and disease (13, 20). Previous studies have investigated the potential contribution of single nucleotide polymorphisms (SNPs) in genes encoding CC-chemokine ligands and receptors to CD outcome. The genetic variants of the *CCL5* gene rs2107538 and rs2280788 SNPs were found to be monomorphic in a Colombian population and did not allow associations with CCC development (21). The *CCR5* variants +59029G allele (rs1799987), −2733G allele (rs2856758), and *CCR5* CC genotype (rs3176763) were associated with a reduced risk of developing CCC in different populations (21–23). However, *CCR5* +59029A > G (rs1799987) did not influence left ventricular systolic dysfunction in patients with Chagas heart disease in a Brazilian cohort (24), and the *CCR5* −1835T allele (rs1800024) was associated with CCC severity in a Colombian population (25). In addition, the *CCL2* −2518 A allele (rs1024611) and +3726AA (rs2530797) variants have been correlated with susceptibility to and/or severity of CCC in cohorts from Brazil and Colombia (23, 26).

Understanding the mechanisms controlling the colonization of heart tissue by inflammatory cells may reveal targetable molecules to modulate the inflammatory response associated with CCC severity and improve the prognosis of CD patients. The few available studies, frequently with a small number of patients and controversial data, underscore the need for studies to comprehend the participation of polymorphisms in CC-chemokine ligands and receptors in the outcome of Chagas heart disease. Hence, we evaluated the potential association of functional gene variants in *CCL2* (rs1024611) and *CCL5* (rs2107538 and rs2280788) and the CCL5 receptors *CCR1* (rs3181077, rs1491961, rs3136672) and *CCR5* (rs1799987) with the risk of developing and the severity of Chagas heart disease in a group of patients in the State of Pernambuco, Northeast Brazil. Furthermore, to gain insight into the contribution of CCL5 and its receptors CCR1 and CCR5 to the pathogenesis of Chagas heart disease, we used an experimental model of CCC (27, 28), CCR5-deficient mice and interventions with the CCR1/CCR5 antagonist Met-RANTES.

## Materials and Methods

### Ethics Statement

This study was carried out in accordance with the recommendations of the Ethics Committees of Fiocruz/RJ (541/09) and PROCAPE/UPE (80210/10). All subjects provided written informed consent in accordance with the Declaration of Helsinki.

This study was carried out in strict accordance with the recommendations of the Guide for the Care and Use of Laboratory Animals of the Brazilian National Council of Animal Experimentation (http://www.sbcal.org.br/) and Federal Law 11.794 (October 8, 2008). The Institutional Committee for Animal Ethics of Fiocruz (CEUA-Fiocruz-L004/09; LW-10/14) approved all experimental procedures used in the present study. All presented data were obtained from three independent experiments (Experiment Register Books #31 and 57, LBI/IOC-Fiocruz).

### Study Population and Diagnostic Criteria

For the human genetics study, a group of 406 patients undergoing surveillance at the Ambulatório de Doença de Chagas e Insuficiência Cardíaca do Pronto Socorro Cardiológico de Pernambuco (PROCAPE)/Universidade do Estado de Pernambuco (UPE) was enrolled for an unmatched association study. At enrollment, 10 mL of peripheral blood was collected for serological diagnosis confirmation and DNA isolation. According to the second Brazilian Consensus on CD (29), the serological diagnosis of CD was determined by at least two independent tests, including enzyme-linked immunosorbent assay (ELISA) and indirect immunofluorescence, performed by the Central Reference Laboratory (LACEN) of Pernambuco, Brazil. Patients under 18 years of age or presenting the digestive and cardio-digestive forms of CD and co-infections were excluded. At baseline, the patients were evaluated by anamnesis, and findings on 12-lead electrocardiography were recorded (ECG; Ecafix, São Paulo, SP, Brazil). Echocardiography (ECHO) Doppler two-dimensional and M-mode imaging was performed using a Vivid 3 (GE Health Care, Wauwatosa, WI, USA) with digitally recorded images. Participants were classified according to the I Latin American Guideline for the Diagnosis and Treatment of Chagas Heart Disease (30).

### DNA Isolation and Genotyping

Genomic DNA was isolated from frozen blood samples using a modified precipitation technique by salting out as previously described (31). After extraction, DNA samples were quantified using a NanoDrop ND-1000 spectrophotometer (NanoDrop Technologies, USA). As described in Table S1 in Supplementary Material, we analyzed seven SNPs using TaqMan^®^ genotyping assays: *CCL2* (rs1024611); *CCL5* (rs2107538 and rs2280788); *CCR1* (rs3181077, rs1491961 and rs3136672); and *CCR5* (rs1799987). Reactions were performed with 30 ng of yield DNA for each sample by following the manufacturer’s recommendations for allelic discrimination in the ViiA™ 7 Real-Time PCR System (Applied Biosystems, USA). Raw genotyping data are deposited at www.arca.fiocruz.br/handle/icict/23621.

### Functional Analysis

Quantification of CCL2 and CCL5 concentrations in serum was performed using ELISA DuoSet kits (R&D Biosystems, Minneapolis, MN, USA) according to the manufacturer’s instructions. Data were analyzed (GraphPad Prism software version 5.0 for Windows) by comparing chemokine levels (i) correlating with left ventricular ejection fraction (LVEF; %), (ii) between different clinical groups, and (iii) by stratifying chemokine levels according to the corresponding SNP genotypes. A group of 20 individuals with triatomine exposure and from the same region as cases with negative serology for *T. cruzi* infection was included as noninfected (NI) controls for functional analysis. Additionally, CCL2 and CCL5 concentrations were determined in supernatants of peripheral blood cell cultures using the CBA kit for human chemokines (BD™, San Diego, CA, USA) according to manufacturer’s recommendations. Data acquisition was performed using CellQuest Pro software and analysis using v3 Array FCAP software (BD™, Franklin Lakes, NJ, USA). The lower detection limits were 2.7 and 1.26 pg/mL for CCL2 and CCL5, respectively.

### Mice, Experimental Infection, and Met-RANTES Treatment

C57BL/6 (H-2^b^, *ccr5*^+/+^) and *Ccr5*-deficient (*Ccr5^tm1Kuz^* on the C57BL/6 background, *Ccr5*^−/−^) mice were originally purchased from Jackson Laboratories (Sacramento, CA, USA). Female mice aged between 4 and 6 weeks were obtained from the Oswaldo Cruz Foundation animal facilities (CECAL, Rio de Janeiro, Brazil) and maintained under specific pathogen-free conditions. In all sets of experiments, 3 to 5 sex- and age-matched NI controls were analyzed per time point in parallel with 5 to 10 infected mice according to the experimental protocol. Mice were infected with the Colombian *T. cruzi* DTU I strain (32) by intraperitoneal injection of 100 or 1,000 blood trypomastigotes. Parasitemia was estimated in 5 µL of tail vein blood. After the peak of parasitemia, detection of rare circulating trypomastigotes marked the onset of the chronic phase of infection, as previously described (33). Groups of 7 to 10 mice were subcutaneously inoculated daily with 0.1 mL of *in vivo* injection-grade saline (BioManguinhos, Rio de Janeiro, RJ, Brazil) or saline containing 10 µg of Met-RANTES from 120 to 150 days postinfection (dpi) (17) and analyzed at 150 dpi. The CCR1/CCR5 partial antagonist Met-RANTES was kindly provided by Dr. Amanda Proudfoot (Serono Pharmaceuticals, Geneva, Switzerland). Parasitemia and survival rates were evaluated weekly. According to the experimental protocols, mice were euthanized under anesthesia at 70, 120, or 150 dpi. The experiments were reproduced two or three times.

### Reagents and Antibodies

For immunohistochemical staining, the monoclonal antibodies anti-mouse CD4 (clone GK1.5) and CD8a (53-6.7) produced in our laboratory (LBI/IOC-Fiocruz, Brazil) and anti-F4/80 (macrophage) purchased from Caltag (Burlingame, CA, USA) were used. Anti-TNF-biotin was purchased from BD PharMingen (San Diego, CA, USA). Purified anti-CCR5 (eBioT21/8) was purchased from Novus Biologicals (Littleton, CO, USA). Purified anti-CCR1 (E05-10) was obtained from Santa Cruz Biotechnology (Dallas, TX, USA). Biotinylated anti-rat immunoglobulin was purchased from Dako (Glostrup, Denmark). Anti-goat Ig-biotin was purchased from Dako (Carpinteria, CA, USA). Anti-mouse Ig-biotin was obtained from Life Science (Rockford, IL, USA). Streptavidin–HPR conjugate was purchased from GE Healthcare (Buckinghamshire, UK). Appropriate controls were prepared by replacing the primary antibodies with purified rat immunoglobulin. For flow cytometry studies using mouse cells, anti-TCR-FITC [H57.597 was purchased from Southern Biotech (Birmingham, AL, USA)]. The anti-Ly6C-APC (HK1.4) and CD11c-PECy7 (N418) antibodies were obtained from eBioscience (San Diego, CA, USA). Anti-IL-10-PE (clone JES5-2A5) was made by Caltag (Burlingame, CA, USA). FITC- or APC-conjugated anti-mouse CD8a (53-6.7), anti-CCR5-PE (HM-CCR5, 7A4), anti-IL-10-APC (JES5-16E3), anti-TNF-PECy7 (MP6-XT22), CD62L-PE (MEL-14), CD11b-PE (M1/70), and anti-CD14-FITC (rmC5-3) were purchased from BD PharMingen (San Diego, CA, USA). Anti-CCR1-PerCp (clone sc-6125) was obtained from Santa Cruz Biotechnology (Dallas, TX, USA). The F4/80-PE-Texas red (BM8) antibody was obtained from Invitrogen (Rockford, IL, USA). To study the expression of CCR1 and CCR5 on human CD8^+^ T cells, we used anti-CD8-FITC (3B5) purchased from Caltag (Burlingame, CA, USA) and anti-CCR1-AF (clone 5354) and anti-CCR5-PE (clone 2D7/CCR5) obtained from BD PharMingen (San Diego, CA, USA). Appropriate controls were prepared by replacing the primary antibodies with their respective isotypes, which were also obtained from BD PharMingen (San Diego, CA, USA) or from Southern Biotech (Birmingham, AL, USA). All the antibodies (1.5–20 µg/mL) and reagents were used according to the manufacturers’ instructions.

### Flow Cytometry Analysis

To phenotype mouse cells, spleens were minced, and red blood cells were removed using lysis buffer (Sigma-Aldrich, St. Louis, MO, USA). For *ex vivo* analysis, splenocytes were incubated with monensin in RPMI 10% fetal bovine serum for 4 h at 37°C and 5% CO_2_ according to the manufacturer’s instructions (BD Golgi Stop™ Cat 554724, San Diego, CA, USA). The cells were collected after 1 h on an ice bath, washed, and resuspended in PBS containing 2% fetal calf serum/2% mouse serum and labeled as previously described (27). For analysis, 100,000 to 300,000 events were acquired with a CyAn-ADP flow cytometer (Beckman-Coulter, Houston, TX, USA). After gating in singlets and exclusion of dead cells, the cell populations were analyzed using Summit v.4.3 Build 2445 software (Dako, Carpinteria, CA, USA), as described elsewhere (27). To identify CD8^+^ T cells, the gating strategy was as follows: singlets (R1), dead-cell exclusion (FSC-A × SSC-Lin, R2), TCR × CD8 dot plot (gating on TCR^+^CD8^+^ cells), CCR1 × CCR5 (gating on single-positive cells), and TNF × IL-10 dot plot (% in quadrants of single-positive cells). To identify CD14^+^ cells, the gating strategy was as follows: singlets (FSC-Lin × FSC-Area, R1), dead-cell exclusion (FSC-A × SSC-Lin, R2), CD14^+^ cells (histogram, R3), CCR1 × CCR5 dot plot (gating on single-positive cells), and TNF × IL-10 dot plot (% in quadrants of single-positive cells). To characterize CD14^+^ cells, we used the following staining strategies: strategy 1 [singlets (FSC-Lin × FSC-Area, R1), dead-cell exclusion (FSC-A × SSC-Lin, R2), CD14 × Ly6C dot plot, and CD14 × CD11c]; strategy 2 [singlets (FSC-Lin × FSC-Area, R1), dead-cell exclusion (FSC-A × SSC-Lin, R2), CD14 × CD45R dot plot (gating on CD14^+^CD45R^+^ cells), and CD11b × F4/80 dot plot].

### Stimulation of Human Peripheral Blood Mononuclear Cells *In Vitro* by *T. cruzi* Antigens and Cell Phenotyping

Peripheral blood from NI individuals and CD patients was stimulated with parasite epimastigote antigens (34), as T cells from chagasic patients respond to epimastigote and trypomastigote lysate with similar strengths (35). For this analysis, 1 mL of heparinized whole peripheral blood was used for culture in RPMI 1640 medium (Sigma-Aldrich, St. Louis, MO, USA) supplemented with l-glutamine, 1% antibiotic (10,000 U of penicillin, 10,000 U of streptomycin Sigma-Aldrich, Louis, MO, USA) and 10% fetal bovine serum (Sigma-Aldrich, St. Louis, MO, USA) in the presence of epimastigote extract (25 µg/mL) or was left unstimulated (controls) in a final volume of 2 mL of culture medium and incubated at 37°C and 5% CO_2_ for 24 h. Next, the supernatants were collected, and the cells were harvested with 20 mM EDTA (Sigma-Aldrich, St. Louis, MO, USA), washed in PBS containing 0.5% bovine serum albumin (Sigma-Aldrich, St. Louis, MO, USA), and labeled as previously described (36). The data were acquired using a FACScalibur flow cytometer (Becton Dickinson, San Jose, CA, USA) and analyzed with CellQuest Pro software.

### Immunohistochemistry

Groups of five infected and three age-matched control mice were sacrificed under anesthesia at 70, 120, and 150 dpi. The hearts of the mice were removed, embedded in tissue-freezing medium (Tissue-Tek, Miles Laboratories, Elkhart, IN, USA), and stored in liquid nitrogen for analysis by immunohistochemistry. Serial 3-µm thick cryostat sections were fixed in cold acetone and subjected to indirect immunoperoxidase staining, as previously described (33, 37). The number of positive CD4^+^, CD8^+^, and F4/80^+^ cells in 100 microscopic fields was counted. The positively stained areas for CCR1, TNF, and IL-10 in 25 microscopic fields (12.5 mm^2^) in two sections per heart tissue were evaluated with a digital morphometric apparatus. The images were digitized using a Sight DS-U3 color-view digital camera adapted to an Eclipse Ci-S microscope and analyzed with NIS Elements BR version 4.3 software (Nikon Co., Tokyo, Japan). According to the analyzed parameters, the data are presented as numbers of parasite nests or cells per 100 microscopic fields or the percent positive area in the heart or spleen tissue (magnification, 400×). The analyses were performed in a blinded manner by an independent reader.

### Creatine Kinase Detection

The activity of the creatine kinase myocardial band (CK-MB) is a marker of myocardial injury associated with CCC severity in experimental models (28, 38–41). CK-MB activity was measured with commercial kits (Kovalent do Brasil, São Gonçalo, RJ, Brazil), as previously described (27). The assay was adapted for reading in a microplate spectrophotometer (ASYS Hitech GmbH, Eugendorf, Austria) to allow the study of small quantities of mouse serum according to the manufacturer’s recommendations. The optical density at 340 nm was recorded every 2 min for 15 min.

### CCL5 and TNF Determination by ELISA in Mouse Heart Tissue

Mouse hearts were harvested, washed to remove blood clots, and weighed. Extracts were prepared after tissue homogenization in PBS (0.3 mL) using the tissue grinder IKA-Ultra Turrax T10 (Sigma-Aldrich, Louis, MO, USA) on an ice bath, and supernatants were collected after centrifugation (3,000 *g*, 10 min, 4°C). The concentrations of CCL5 and TNF in the cardiac tissue extracts were evaluated with an ELISA DuoSet kit (R&D Systems, Minneapolis, MN, USA) according to the manufacturer’s instructions. Diluted (1:2, 1:10) tissue extracts were analyzed in duplicate. Standards consisted of ½log dilutions of the recombinant cytokines from 1 pg/mL to 100 ng/mL. This ELISA method consistently detects concentrations above 10 pg/mL. Data were normalized considering the heart weight, and results are expressed as CCL5 and TNF concentrations in 100 mg of heart tissue.

### Statistical Analysis

Genetic analyses were performed in R environment version 3.3.3 with the following packages: coin, epiDisplay, gap, genetics, haplo.stats, and SNPassoc. Comparisons among demographic or clinical variables were performed either by the chi-square test or Kruskal–Wallis test when appropriate. The association of genotypic and minor allele carrier frequencies with CD was evaluated by unconditional logistic regression comparing individuals between the different stages of the cardiac form. Two nongenetic variables, gender and ethnicity, were included as categorical covariates in the regression model. First, we sought to determine the SNP influence on development of the cardiac form of CD. For this analysis, patients from the A group were considered as controls, while patients from the B1 and C groups were referred to as cases. Then, to examine the influence of SNPs on the severity of CD cardiomyopathy, group B1 (mild CCC) was tested as the control group and group C (severe CCC) as the case group. Additionally, allele-dose effects were tested with the Cochran–Armitage trend test. Statistical significance was considered for *P*-values < 0.05. For polymorphisms located within the same gene, haplotype frequencies were estimated by maximum-likelihood and compared using the same unconditional regression models as the analysis for the individual SNPs.

The sample size was determined based on the experience of our group and previous studies using the model of experimental CCC. Therefore, no formal sample size was calculated. Data are expressed as the arithmetic mean ± SE. For statistical analyses, we used the Student’s *t* test to compare two groups. Comparisons between groups were carried out by analysis of variance followed by the Bonferroni *post hoc* test. All statistical tests were performed with GraphPad Prism 5.0 (La Jolla, CA, USA). Differences were considered statistically significant when *P* < 0.05.

## Results

### Epidemiological and Clinical Characterization of the CD Patients

The 406 patients enrolled in this study were classified, as previously described (30), as stage A (*n* = 110), without cardiac symptoms and with normal ECG and ECHO; stage B1 (*n* = 163), with structural heart disease, evidenced by ECG or ECHO, but with normal global ventricular function and neither current nor previous signs and symptoms of CHF; and stage C (*n* = 133), with ventricular dysfunction and current or previous symptoms of CHF. Mean ages for the groups were 51 ± 12 years (group A), 60 ± 13 years (group B1) and 60 ± 11 years (group C). Independently of the studied group, a higher frequency of females was observed (69.5%), and most of the patients identified themselves as mestizo (68.3%). A monthly income of up to one minimum wage (~US$ 300; 66–72%) and an education level of up to 4 years (80–88%) were observed for most patients. As an important clinical variable, LVEF was similar in patients in group A (67 ± 5%) and group B1 (66 ± 6%) but showed a significant decrease in the patients in group C (40 ± 11%, *P*-value < 0.001 vs A and B1). The use of the trypanocidal drug benznidazole was registered in 44% of stage A, 12% of stage B1, and 14% of stage C patients (42).

### The rs2107538 SNP at *CCL5* Is Associated With Protection Against the Cardiac Form of CD

For the genotyping study, all polymorphisms had a call rate efficiency of 95%. The *CCL2* rs1024611 gene variant was not a genetic marker for CCC in our group of patients An SNP (Table 1). The study of the *CCL5* SNP rs2280788 (−28G > C) revealed a low frequency of the mutant allele in this population, which did not allow a study of association with the risk or severity of CCC (Table 1). However, an association of *CCL5* −403C > T (rs2107538) with protection against developing CCC was observed for genotype CT (OR = 0.5, *P*-value = 0.04) and T carriers (OR = 0.5, *P*-value = 0.01) compared to patients without cardiopathy as the control group (A) and cardiopathic patients with CHF symptoms as the case group (C), as shown in Table 1. The analysis of *CCR1* SNPs (rs3181077, rs1491961, and rs3136672) and of the *CCR5* variant rs1799987 showed no significant associations with CCC outcome (Table 2). Furthermore, the haplotype analysis performed for *CCL2*–*CCL5* (Table S2 in Supplementary Material) and *CCR1*–*CCR5* (Table S3 in Supplementary Material) clusters showed no association with susceptibility to or severity of CCC.

**Table 1:** Analysis of single nucleotide polymorphisms located in the *CCL2* and *CCL5* genes.

				A vs B1		A vs C		B1 vs C	
	Stage A *N* = 110	Stage B1 *N* = 163	Stage C *N* = 133	OR^a^ [95% CI]	*P*-value	OR^a^ [95% CI]	*P*-value	OR^a^ [95% CI]	*P*-value
***CCL2*-2518 rs1024611**									
AA	46 (0.51)	60 (0.43)	56 (0.49)			Reference			
AG	33 (0.36)	63 (0.45)	48 (0.42)	1.5 [0.9–2.8]	0.31	1.1 [0.6–2.1]	0.62	0.8 [0.5–1.3]	0.57
GG	12 (0.13)	16 (0.12)	11 (0.09)	1.1 [0.4–2.6]		0.7 [0.3–1.9]		0.7 [0.3–1.7]	
G carriers	45 (0.49)	79 (0.57)	59 (0.51)	1.4 [0.8–2.5]	0.20	1.1 [0.6–1.8]	0.85	0.8 [0.5–1.3]	0.30

***CCL5*-403 rs2107538**									
CC	38 (0.43)	74 (0.54)	68 (0.60)			Reference	–		
CT	42 (0.48)	57 (0.42)	36 (0.32)	0.7 [0.4–1.2]	0.11	0.5 [0.2–0.8]	**0.04**	0.7 [0.4–1.2]	0.24
TT	8 (0.09)	6 (0.04)	9 (0.08)	0.3 [0.1–1.0]		0.6 [0.2–1.6]		1.6 [0.5–4.9]	
T carriers	50 (0.57)	63 (0.46)	45 (0.40)	0.6 [0.4–1.1]	0.10	0.5 [0.3–0.8]	**0.01**	0.8 [0.5–1.3]	0.38

***CCL5*-28 rs2280788**									
GG	33 (0.97)	114 (0.97)	85 (0.98)	56 (0.49)			Reference			
GC	1 (0.03)	4 (0.03)	2 (0.2)	0.8 [0.08–8.5]	0.88	0.8 [0.06–9.6]	0.85	0.7 [0.1–4.0]	0.66

*^a^Odds ratio (OR) values shown are corrected for gender and ethnicity*.

*Results are shown as *n* (frequency)*.

*CI, confidence interval*.

*Total genotype counts can vary due to different genotypic call rates*.

*Bold font is used to highlight the different SNPs, the clinical stages of the patients and the comparisons that were performed. Also, significant p-values are in bold*.

**Table 2:** Analysis of single nucleotide polymorphisms located in the *CCR1* and *CCR5* genes.

				A vs B1		A vs C		B1 vs C	
	Stage A *N* = 110	Stage B1 *N* = 163	Stage C *N* = 133	OR^a^ [95% CI]	*P*-value	OR^a^ [95% CI]	*P*-value	OR^a^ [95% CI]	*P*-value
***CCR1*rs3181077**									
TT	70 (0.76)	101 (0.74)	75 (0.67)	Reference					
CT	20 (0.22)	35 (0.25)	33 (0.29)	1.3 [0.7–2.4]	0.46	1.6 [0.8–3.1]	0.29	1.3 [0.7–2.3]	0.17
CC	2 (0.02)	1 (0.01)	4 (0.04)	0.3 [0.03–3.8]		1.7 [0.3–9.7]		5.9 [0.6–55.0]	
C carriers	22 (0.24)	36 (0.26)	37 (0.33)	1.2 [0.6–2.2]	0.58	1.6 [0.9–3.1]	0.12	1.4 [0.8–2.4]	0.24

***CCR1*rs1491961**									
CC	70 (0.76)	102 (0.73)	77 (0.68)	Reference					
CT	20 (0.22)	36 (0.26)	33 (0.29)	1.3 [0.7–2.5]	0.43	1.6 [0.8–3.1]	0.32	1.2 [0.7–2.1]	0.20
TT	2 (0.02)	1 (0.07)	4 (0.04)	0.3 [0.03–3.7]		1.7 [0.3–9.5]		5.7 [0.6–53.0]	
T carriers	22 (0.24)	37 (0.27)	37 (0.33)	1.2 [0.6–2.2]	0.55	1.6 [0.9–3.0]	0.13	1.3 [0.8–2.3]	0.31

***CCR1*rs3136672**									
GG	70 (0.76)	94 (0.68)	74 (0.65)	Reference					
AG	20 (0.22)	39 (0.28)	32 (0.28)	1.6 [0.8–3.0]	0.23	1.5 [0.8–2.8]	0.13	1.0 [0.6–1.8]	0.83
AA	2 (0.02)	6 (0.04)	8 (0.07)	2.4 [0.4–12.4]		3.8 [0.8–19.1]		1.4 [0.4–4.5]	
A carriers	22 (0.24)	45 (0.32)	40 (0.35)	1.7 [0.9–3.1]	0.10	1.7 [0.9–3.1]	0.10	1.1 [0.6–1.8]	0.82

***CCR5*+59029 rs1799987**									
AA	24 (0.27)	35 (0.25)	32 (0.28)	Reference					
AG	43 (0.49)	70 (0.51)	55 (0.49)	1.0 [0.5–2.0]	0.96	0.9 [0.5–1.8]	0.96	0.9 [0.5–1.7]	0.94
GG	21 (0.24)	33 (0.24)	26 (0.23)	1.1 [0.5–2.4]		0.9 [0.4–2.0]		0.9 [0.4–1.8]	
G carriers	64 (0.73)	103 (0.75)	81 (0.72)	1.1 [0.6–2.0]	0.83	0.9 [0.5–1.7]	0.77	0.9 [0.5–1.6]	0.80

*^a^ Odds ratio (OR) values shown are corrected for gender and ethnicity*.

*Results are shown as *n* (frequency)*.

*CI, confidence interval*.

*Total genotype counts can vary due to different genotypic call rates*.

### CCL5 Serum Levels Are Enhanced in CD Patients but Are Not Related to CCC Severity or CCL5 Genotype

**Figure 1:**
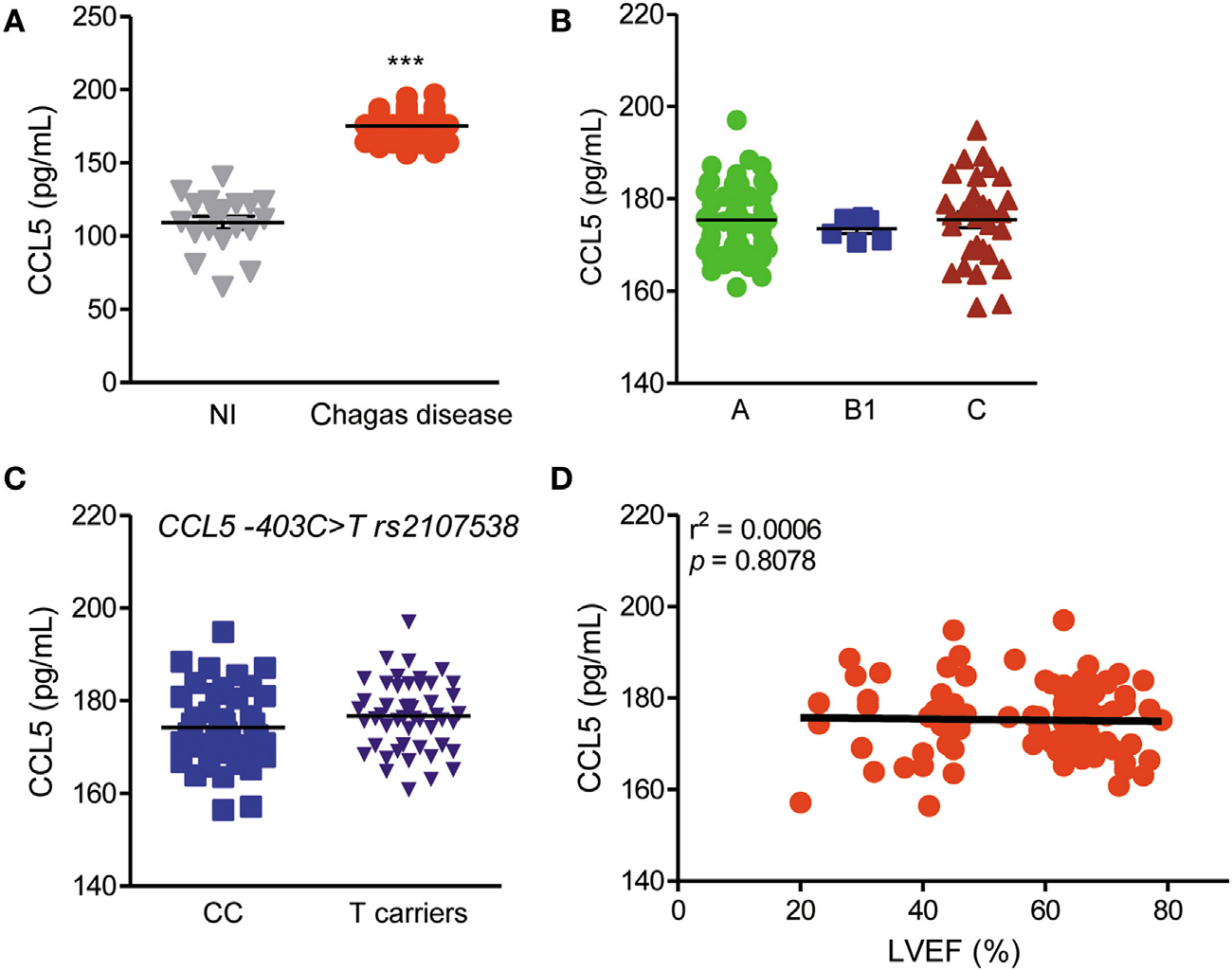
CCL5 levels and association with disease severity, genotypes and left ventricular ejection fraction (LVEF) in *T. cruzi*-infected patients. **(A)** CCL5 levels in the serum of seronegative noninfected (NI) individuals and individuals who were seropositive for Chagas disease (CD); ***P < 0.001 (Student’s *t*-test). **(B)** CCL5 concentrations in the serum of CD patients grouped as A (asymptomatic), B1 [mild chronic chagasic cardiomyopathy (CCC)], or C (severe CCC) (analysis of variance, Bonferroni posttest). **(C)** Comparison of CCL5 serum levels according to the absence (CC) or presence of rs2107538 allele T (Mann–Whitney test). **(D)** Correlation between the CCL5 serum concentrations and LVEF in patients; *P* = 0.8078, *r*^2^ = 0.0006 (linear regression).

CCL5 serum levels were enhanced in CD patients compared with those in NI residents in endemic areas (Figure 1A), but these levels were not associated with the risk or severity of CCC (Figure 1B). Additionally, CCL5 serum levels were not associated with genotypes of the *CCL5* −403C > T rs2107538 variant (Figure 1C) or *CCR5* (rs1799987) and *CCR1* (rs1491961, rs3136672, rs3181077) gene variants (Figure S1 in Supplementary Material). In the studied group, CCC severity was associated with a reduction in LVEF, as previously shown (42). However, there was no correlation between CCL5 serum concentrations and LVEF values (Figure 1D). In comparison to NI matched controls, CCL2 serum concentrations were also increased in CD patients but were not associated with disease severity, genotype, or loss of LVEF (Figure S2 in Supplementary Material).

### CCL5 Levels Are Increased in Heart Tissue, and an Altered Balance of CCR1/CCR5 Expression on CD8^+^ T Cells Is Detected in Chronically *T. cruzi*-Infected Mice

**Figure 2:**
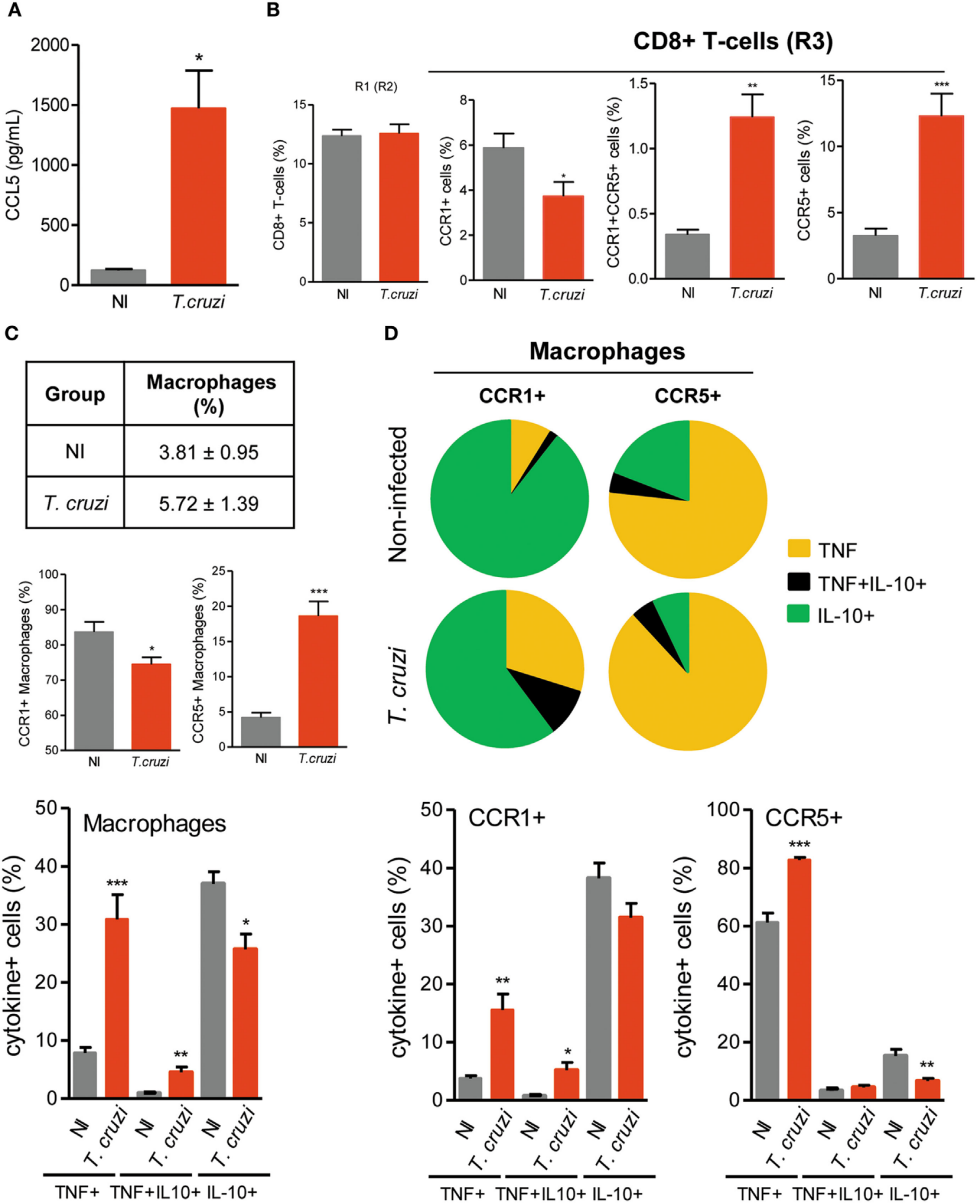
CCL5 levels and CCR1 and CCR5 expression in CD8^+^ T cells and CD14^+^ macrophages of *T. cruzi*-infected C57BL/6 mice. Mice were infected with 100 trypomastigote forms of the Colombian *T. cruzi* strain and analyzed at 120 days postinfection. The heart was removed, proteins were extracted, and CCL5 concentrations were estimated by enzyme-linked immunosorbent assay. Splenocytes were collected and stained for intracellular cytokines (TNF, IL-10) and surface cell markers (CD8, CD14, CCR1, CCR5). **(A)** CCL5 levels in the heart tissue extracts of *T. cruzi*-infected mice compared to noninfected (NI) controls. **(B)** Percentages of total CD8^+^ T cells and CD8^+^ T cells expressing CCR1 and/or CCR5 in the spleen of NI and *T. cruzi*-infected mice. **(C)** The table shows percentages of total CD14^+^ macrophages. Graphs show the frequencies of CD14^+^ macrophages expressing CCR1 or CCR5 and CD14^+^ macrophages expressing TNF, TNF/IL-10, or IL-10 in the spleen of NI and *T. cruzi*-infected mice. **(D)** Pie charts represent the fractions of CCR1^+^ or CCR5^+^ CD14^+^ macrophages carrying each of the intracellular cytokine phenotypes shown in the legend. Graphs show the frequencies of CCR1^+^ or CCR5^+^ CD14^+^ macrophages expressing TNF, TNF/IL-10 or IL-10. Data represent two independent experiments with three NI and five to seven infected mice [*P < 0.05, **P < 0.01, ***P < 0.001, *T. cruzi*-infected mice vs NI (Student’s *t*-test)].

To shed light on the contribution of CCL5 and its receptors CCR1 and CCR5 in Chagas heart disease, we used a model of CCC that presents fibrosis, CD8-enriched myocarditis, myocardial injury and electrical and functional abnormalities (27, 28, 41). An increased concentration of CCL5 was detected in the heart tissue of chronically (120 dpi) *T. cruzi*-infected mice (Figure 2A). In the spleen, most of the CD8^+^ cells were T cells (Figure S3A in Supplementary Material). The frequencies of CD8^+^TCR^+^ cells were similar in splenocytes of NI and chronically *T. cruzi*-infected mice (Figure 2B). However, in infected mice, an altered balance of CCR1 and CCR5 expression was achieved, with a significant reduction in CCR1^+^ and an increase in CCR1^+^CCR5^+^ and CCR5^+^ cell frequencies among CD8^+^ T cells (Figure 2B). Additionally, the increase in the frequencies of CCR5^+^ CD8^+^TCR^+^ cells showed a moderate negative correlation (*r*^2^ = 0.424) with the decreased frequencies of CCR1^+^ CD8^+^TCR^+^ cells (Figure S3B in Supplementary Material).

Regarding the inflammatory/regulatory profiles, in NI sex- and age-matched control mice, CCR1^+^CD8^+^ T cells showed a high frequency of IL-10^+^ cells, whereas CCR5^+^ CD8^+^ T cells presented a more balanced TNF/IL-10 profile (Figure S4 in Supplementary Material). In chronically *T. cruzi*-infected mice (120 dpi), TNF^+^ cells predominated in both the CCR1^+^ and CCR5^+^ CD8^+^ T-cell populations, while CCR5^+^ cells presented a higher frequency of TNF^+^ cells compared with CCR1^+^ CD8^+^ T cells. Moreover, the TNF/IL-10 ratio was higher in CCR5^+^ compared with CCR1^+^ CD8^+^ T cells of *T. cruzi*-infected mice (Figure S4 in Supplementary Material).

### Antagonist IL-10 and TNF Phenotypes Are Sustained in CCR1^+^ and CCR5^+^ Macrophages of Chronically *T. cruzi*-Infected Mice

The frequencies of splenic CD14^+^ macrophages remained similar in *T. cruzi*-infected mice compared with those in NI controls (Figure 2C). In NI and *T. cruzi*-infected mice, the CD14^+^ cells were mostly CD11c^neg^, Ly6C^neg^, and CD45R^+^F4/80^+^, either CD11b^+^ or CD11b^neg^ (Figures S5A,B in Supplementary Material). In comparison to NI age-matched controls, in *T. cruzi*-infected mice, the numbers of splenic CD14^+^ cells were increased (Figure S5C in Supplementary Material). Although CCR1^+^CD14^+^ macrophages predominated in NI and infected mice, a significant decrease in the percentage of CCR1^+^ and an increase in the percentage of CCR5^+^ macrophages were detected in *T. cruzi*-infected mice (Figure 2C). Considering the expression of regulatory IL-10 and inflammatory TNF cytokines, the analysis of single cells revealed that most of the CD14^+^ macrophages were IL-10^+^ in NI mice, whereas there was a significant increase in the percentage of macrophages expressing TNF in *T. cruzi*-infected mice (Figure 2C). Importantly, in NI mice, CCR1^+^ and CCR5^+^ CD14^+^ macrophages presented segregated and antagonist cytokine profiles (Figure S6 in Supplementary Material). In NI mice, CCR1^+^ macrophages were mainly IL-10^+^, whereas CCR5^+^ macrophages were mostly TNF^+^ (Figure 2D). In chronically *T. cruzi*-infected mice, although the percentages of TNF^+^ cells were increased among CCR1^+^ and CCR5^+^ macrophages, the majority of CCR1^+^ macrophages remained IL-10^+^, and most of the CCR5^+^ CD14^+^ macrophages expressed TNF (Figure 2D).

### Depletion of CCR5 Is Beneficial for Myocardial Injury in Chronically *T. cruzi*-Infected Mice

**Figure 3:**
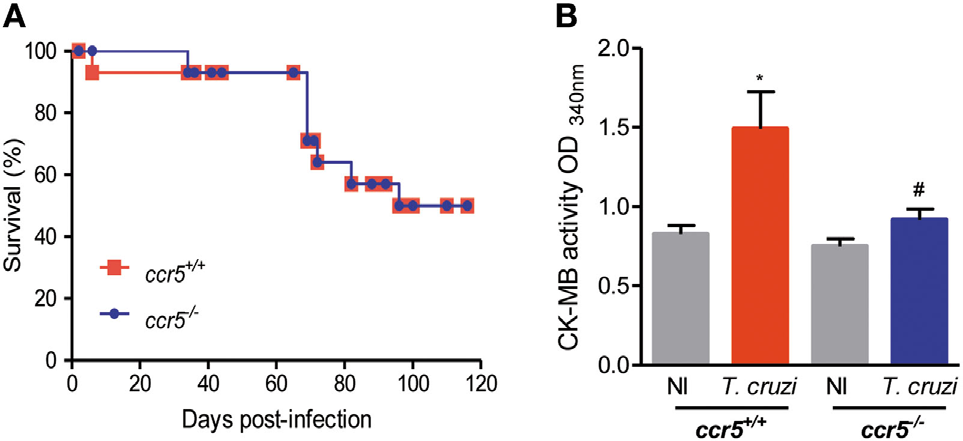
Effects of CCR5 deficiency on survival and myocardial injury in *T. cruzi*-infected mice. Mice were infected with 1,000 trypomastigote forms of the Colombian *T. cruzi* strain and analyzed at 70 days postinfection. **(A)** Survival curves of *T. cruzi*-infected *Ccr5*^+^/^+^ and *Ccr5*^−/−^ mice. Deaths were registered weekly. **(B)** Sera were collected, and CK-MB activity was determined by biochemical assay. Data represent two independent experiments with three noninfected (NI) and five to seven infected mice [*P < 0.05, *T. cruzi*-infected *Ccr5*^+/+^ mice vs NI; ^#^*P* < 0.05, *Ccr5*^−/−^ vs *Ccr5*^+/+^ *T. cruzi*-infected mice (analysis of variance, Bonferroni posttest)].

To study the contribution of the CCL5–CCR5 axis to heart tissue lesions, we infected CCR5-deficient mice. Interestingly, when infected with 1,000 trypomastigote forms of the Colombian strain, *Ccr5*^+/+^ and *Ccr5*^−/−^ mice showed similar survival ratios (Figure 3A). At 70 dpi, *T. cruzi*-infected *Ccr5*^−/−^ mice showed reduced myocardial damage, as assessed by CK-MB activity in serum, in comparison to *Ccr5*^+/+^ infected mice (Figure 3B). Reduced numbers of circulating parasites and a lower heart tissue parasite load, but increased heart inflammation composed of CD4^+^, CD8^+^, and F4/80^+^ cells, were detected in *T. cruzi*-infected *Ccr5*^−/−^ compared with C*cr5*^+/+^ mice (Figure S7 in Supplementary Material), suggesting that the quality rather than the intensity may explain the contribution of heart tissue infiltrating cells to tissue damage.

**Figure 4:**
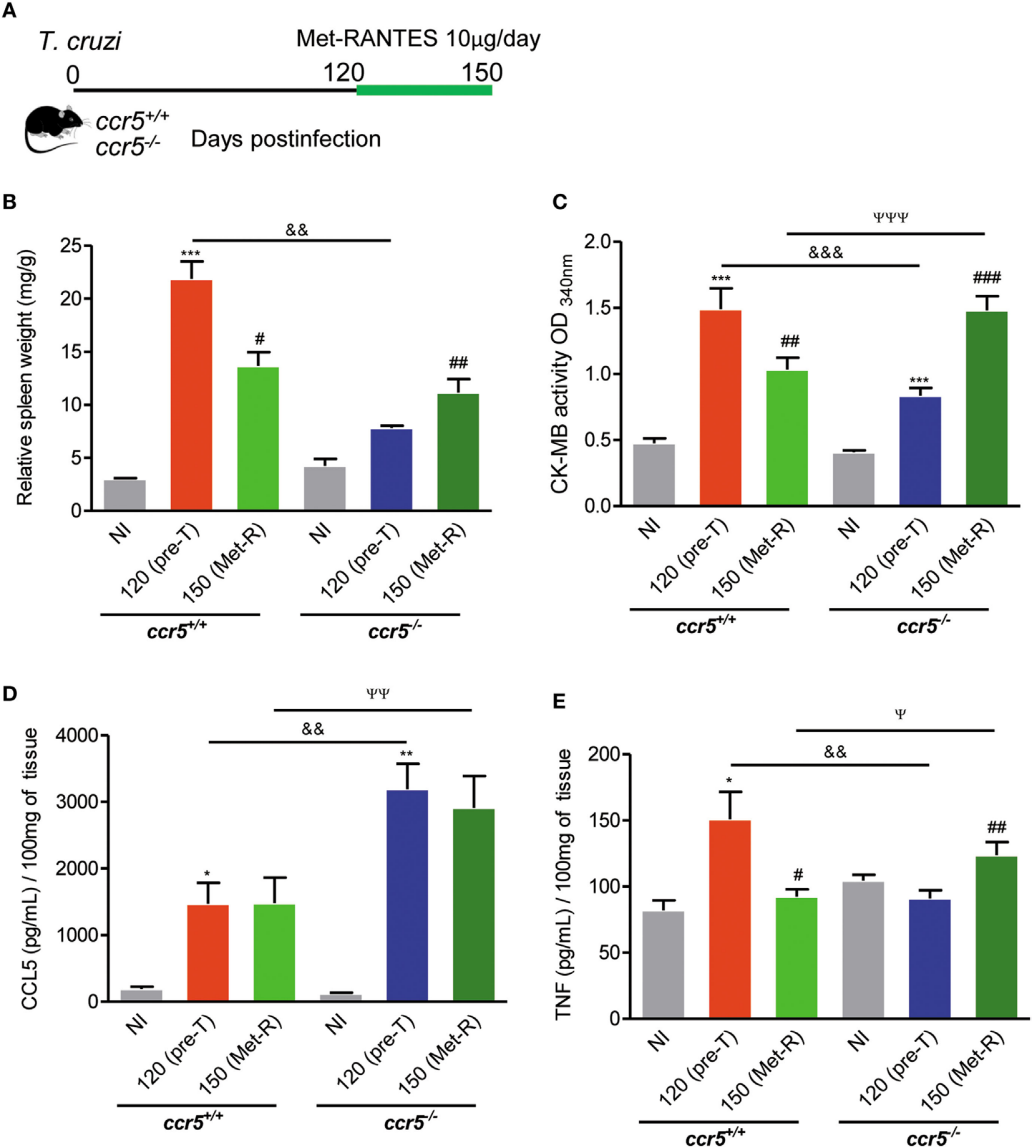
Effects of Met-RANTES treatment on splenomegaly, myocardial injury, and CCL5 and TNF concentrations in the heart tissue of *T. cruzi*-infected mice. **(A)** Mice were infected with 100 trypomastigote forms of the Colombian *T. cruzi* strain and treated with Met-RANTES (Met-R, 10 µg/mice) from 120 to 150 days postinfection (dpi). Sera were collected (at 120 and 150 dpi) for estimation of CK-MB activity. Hearts were collected (at 150 dpi) for evaluation of CCL5 and TNF concentrations by enzyme-linked immunosorbent assay (ELISA). **(B)** The relative weight of the spleen (spleen weight in milligrams/body weight in grams) at 150 dpi. **(C)** CK-MB activity in serum determined by biochemical assay at 120 (pretherapy) and 150 dpi (posttherapy, Met-R). **(D)** CCL5 concentrations in extracts of heart tissue evaluated by ELISA at 150 dpi. **(E)** TNF concentrations in extracts of heart tissue evaluated by ELISA at 150 dpi. Data represent two independent experiments with 3–5 noninfected (NI) and 7–10 infected mice.*P < 0.05, **P < 0.01, ***P < 0.001, *T. cruzi*-infected mice vs NI; ^#^*P* < 0.05, ^##^*P* < 0.01, and ^###^*P* < 0.001, pretreated vs Met-R-treated; ^&&^*P* < 0.01, ^&&&^*P* < 0.001, *Ccr5*^−/−^ vs *Ccr5*^+/+^ *T. cruzi*-infected mice pretreatment (120 dpi); ^ψ^*P* < 0.05, ^ψψ^*P* < 0.01, ^ψψψ^*P* < 0.001, *Ccr5*^−/−^ vs *Ccr5*^+/+^ *T. cruzi*-infected mice posttreatment (150 dpi) with Met-R (analysis of variance, Bonferroni posttest).

CCL5 signals *via* CCR1, CCR3, and CCR5 in mice (11). When *Ccr5*^+/+^ and *Ccr5*^−/−^ mice were infected with 100 trypomastigote forms, all the animals survived, and chronic infection was established. At 120 dpi, the protective effect of CCR5 depletion on myocardial injury was also detected (Figure S8A in Supplementary Material). Furthermore, at this time point of infection, *Ccr5*^+/+^ and *Ccr5*^−/−^ mice showed similar CCR1 expression levels in heart tissue and spleen (Figure S8B in Supplementary Material). To address the differential role of CCR5 and CCR1 in heart tissue lesions, chronically *T. cruzi*-infected *Ccr5*^−/−^ mice were treated with Met-RANTES, a selective antagonist for CCR1 and CCR5, but not CCR3, in mice (43), as schematically shown (Figure 4A). The treatment of chronically *T. cruzi*-infected *Ccr5*^+/+^ mice with Met-RANTES partially reduced *T. cruzi*-elicited splenomegaly (Figure 4B) and the cardiac biomarker of serum CK-MB activity (Figure 4C) at 150 dpi. Nevertheless, treatment of *T. cruzi*-infected *Ccr5*^−/−^ mice with Met-RANTES reversed the beneficial effect of CCR5 depletion on splenomegaly (Figure 4B) and myocardial injury (Figure 4C). At 120 dpi, a significant increase in CCL5 concentrations was observed in the heart tissue of chronically *T. cruzi*-infected *Ccr5*^−/−^ mice compared with *Ccr5*^+/+^-infected mice. Met-RANTES treatment did not affect the CCL5 concentrations in the heart tissue of either *Ccr5*^+/+^ or *Ccr5*^−/−^-infected mice (Figure 4D).

Immunohistochemical characterization revealed that when compared with the respective controls pretherapy, Met-RANTES therapy reduced TNF expression in the heart tissue of *Ccr5*^+/+^ *T. cruzi*-infected mice but increased TNF expression in the heart tissue of infected *Ccr5*^−/−^ mice (Figure S9 Supplementary Material). At 120 dpi (pretherapy), in comparison to age-matched NI mice, elevated TNF concentrations were detected in the heart extracts of *T. cruzi*-infected C*cr5*^+/+^ mice (Figure 4E). Interestingly, Met-RANTES therapy reduced TNF concentrations in the heart tissue of *Ccr5*^+/+^ mice at 150 dpi (Figure 4E). At 120 dpi, compared to *Ccr5*^+/+^ mice, *T. cruzi*-infected *Ccr5*^−/−^ mice presented reduced TNF levels in cardiac tissue (Figure 4E). Conversely, after Met-RANTES treatment, increased TNF concentrations were detected in the heart tissue of *Ccr5*^−/−^ mice (Figure 4E).

### CD8^+^CCR1^+^ T-Cell Frequencies and CCL5 Production Are Increased After *T. cruzi* Antigen Stimulation of Human Peripheral Blood Mononuclear Cells

**Figure 5:**
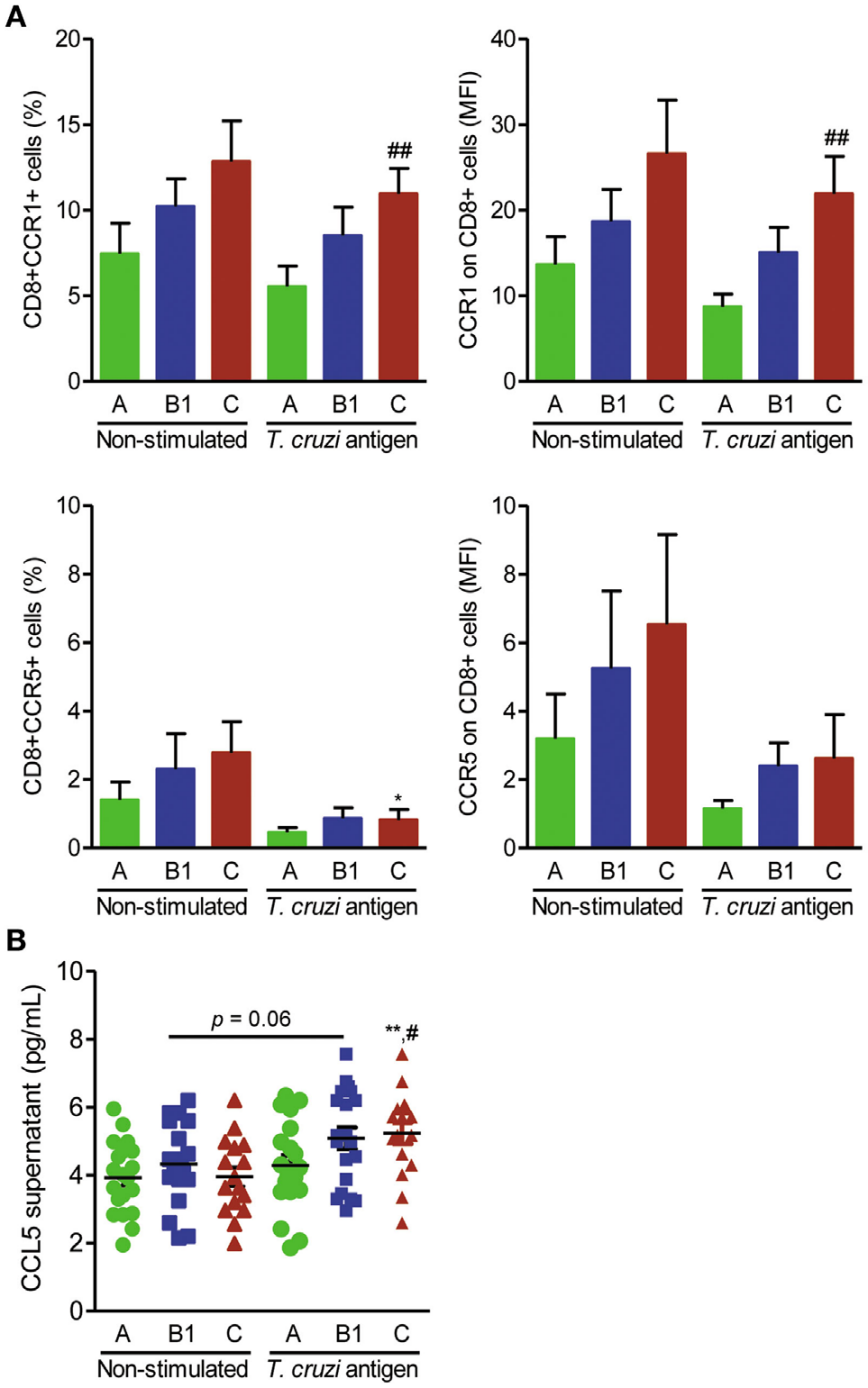
Expression of CCR1^+^ and CCR5^+^ on CD8^+^ T cells and CCL5 production after antigen stimulation in association with the severity of Chagas heart disease. Peripheral blood cells of Chagas disease (CD) patients grouped as A (asymptomatic, *n* = 20), B1 [mild chronic chagasic cardiomyopathy (CCC), *n* = 20], or C (severe CCC, *n* = 16) were collected and stimulated with *T. cruzi* antigen for 24 h. Cells and supernatants were harvested and analyzed for cell markers by flow cytometry and CCL5 production by enzyme-linked immunosorbent assay. **(A)** Frequencies of CCR1^+^ and CCR5^+^ cells and mean fluorescence intensity (MFI) of CCR1 and CCR5 on CD8^+^ T cells; ^##^*P* < 0.01, antigen-stimulated cells from patients in group C vs group A [analysis of variance (ANOVA), Bonferroni posttest]. **(B)** CCL5 concentrations in supernatants of cell cultures of CD patients grouped as A, B1, and C; **P < 0.01, antigen-stimulated vs nonstimulated cells from patients in group C (ANOVA, Bonferroni posttest); ^##^*P* < 0.01, antigen-stimulated cells from patients of group C vs group A (ANOVA, Bonferroni posttest).

The previous findings led us to propose that activation of the CCL5–CCR1 axis, but not CCR5, may trigger a beneficial immune response. To test the feasibility of differential activation of the CCL5–CCR1 axis, peripheral blood mononuclear cells of CD patients were stimulated *in vitro* with parasite antigens and immunophenotyped, and CCL5 levels were measured in the supernatants. The frequencies of CD8^+^CCR1^+^, but not of CD8^+^CCR5^+^, T cells were preserved or even increased among parasite antigen-stimulated blood cells of CD patients (Figure 5A). Additionally, the expression density of CCR1, but not CCR5, was upregulated on CD8^+^ T cells from patients with severe Chagas heart disease after antigen stimulation (Figure 5A). Increased CCL2 concentrations were detected in the supernatants of antigen-stimulated cells compared with those of nonstimulated cells from patients in groups A, B1, and C (Figure S2E in Supplementary Material). Moreover, increased CCL5 concentrations were detected in the supernatants of antigen-stimulated peripheral blood cells of patients with severe Chagas heart disease (group C) compared with those of nonstimulated cells and cells of patients in group A (Figure 5B).

## Discussion

The present study was carried out in CD patients from an endemic region of Northeast Brazil with epidemiological, social, and economic indicators common to populations exposed to *T. cruzi* infection in Latin America (44). In this group of patients, *CCL5* −403C > T (rs2107538) CT heterozygotes and T carriers were associated with protection against heart disease, whereas other variants at the *CCL2, CCL5, CCR1*, and *CCR5* genes were not associated with the outcome of Chagas heart disease in this population. To shed light on the role of CCL5, signaling *via* CCR1 and CCR5, in *T. cruzi* infection, we used an experimental model of CCC. In chronically *T. cruzi*-infected mice, splenic CCR1^+^CD8^+^ T cells were less inflammatory than CCR5^+^CD8^+^ T cells regarding the TNF/IL-10 balance. Moreover, splenic CD14^+^ macrophages showed segregated and antagonist CCR1^+^IL-10^+^ and CCR5^+^TNF^+^ phenotypes. The findings in CCR5-deficient mice supported the conclusion that CCR5^+^ cells play a role in heart tissue damage. Additionally, selective blockade of CCR1 in CCR5-deficient mice supported a protective role for CCR1^+^ cells in CCC. Finally, *T. cruzi* antigen stimulation of human CD8^+^ T cells upregulated CCR1 expression and CCL5 production, supporting the conclusion that the putatively beneficial CCL5–CCR1 axis may be repositioned in CD patients.

Myocarditis, which involves inflammatory CD4^+^ and CD8^+^ T cells and macrophages, is associated with severe Chagas heart disease (4, 5). Therefore, it is reasonable to propose that CC-chemokines, which mainly coordinate mononuclear cell migration to injured tissues (11), play a pivotal role in heart colonization by inflammatory cells in cardiopathic CD patients (6). Consistent with the major accumulation of inflammatory macrophages and T cells, CCL2 and CCL5 mRNA levels were upregulated and CCR2^+^, CCR5^+^, and CCL5^+^ cells were detected in the heart tissue of cardiopathic patients (45, 46). These data support studies attempting to associate functional genetic variants of CC-chemokine ligands and receptors with the risk for and severity of CCC. In this scenario, our data do not support a role for the *CCL2* SNP rs1024611 in susceptibility to and/or severity of CCC. This finding is discordant with previous data showing that the *CCL2* −2518 A allele, which is associated with a low transcriptional level, correlated with susceptibility to CCC in Brazilian case–control association studies with patients born and raised in São Paulo, Minas Gerais and Bahia (23, 26). The *CCL5* SNPs rs2107538 and rs2280788 were found to be monomorphic in a Colombian study and did not allow analysis of the influence of these markers in CCC (21). In our group of CD patients born and residing in Northeast Brazil, the frequency of the minor allele of the *CCL5* variant rs2280788 (−28G > C) was very low. However, an association of *CCL5* rs2107538 (−403C > T) with protection against the development of CCC was observed for CT heterozygotes and T carriers, when cardiopathic with CHF patients (C group) were compared with non-cardiopathic (A group) patients. Case–control studies associated the *CCL5* rs2107538 (403 C > T) with both increased risk of (47) and protection against (48) the development of coronary diseases. One possible explanation for the divergence of these associations can be attributed to the ethnic differences in the distribution of the minor allele of this variant. In this sense, data from a study including five different populations (49) described the variation in the frequency of the mutant allele for this gene variant, which was 15% in American Caucasians and 43% in Americans of African descent. These data reinforce the need to replicate the findings of the present study in independent and ethnically distinct populations of CD patients, as well as to extend the analysis to other variants of the *CCL5* gene, allowing assessments at the haplotype level. Regarding the functional effect of *CCL5* (−403C > T), the *CCL5* rs2107538 T allele has been associated with the upregulation of transcription levels of CCL5 (49). However, another study showed that the −403T allele is not able to influence the transcription levels of the *CCL5* gene (50). In the present study, the association of the *CCL5* rs2107538 −403 gene variant with protection against CCC relied on heterozygote CT and T allele carriers; however, the levels of CCL5 in serum were not correlated with the genotypes. Therefore, to explain the absence of an association of genotype and CCL5 levels in CD patients, one may propose the idea that the −403T single point mutation is not sufficient to overcome other influences controlling CCL5 expression in CD patients. Additionally, one cannot rule out a rapid consumption of this chemokine, considering the increase in expression of CC-chemokine receptors such as CCR5 on inflammatory cells of CD patients (15, 51). Regardless, one should also consider that a genetic polymorphism may not act independently and that multiple changes in a gene may differentially influence the expression and biological features of a molecule. Furthermore, gene expression levels depend on the type of cell in which the gene is expressed and the signals leading to expression.

The increased concentrations of CCL2 and CCL5 in the serum of *T. cruzi*-infected patients compared with NI residents in the same endemic areas of the State of Pernambuco were not associated with disease outcome or, particularly, with LVEF. Conversely, elevated CCL2 concentrations in the serum of CD patients correlated with worsening cardiac function in the population of Minas Gerais, Southeast Brazil (14). CCL5 concentrations in serum, however, had not been previously addressed in chagasic patients. Regarding the biological role of CC-chemokines, *in vitro* CCL2 was shown to participate in parasite uptake and killing in mouse macrophages (52). In addition, CCL2 activates CD8^+^ T cells and macrophages and controls cell migration in experimental acute *T. cruzi* infection (19). Notably, CCL5 has been shown to control parasite growth in human macrophages via induction of nitric oxide (18) and to be essential for parasite burden control in experimental *T. cruzi* oral infection (53). Conversely, CC-chemokines, such as CCL5, signaling *via* CCR1/CCR5 have been implicated in the pathophysiology of experimental CCC, fueling cell migration and heart tissue infiltration of nonbeneficial CD8^+^ T cells (16, 17, 27).

CCL5 signals throughout the CC-chemokine receptors CCR1, CCR3, and CCR5 (11). In the present study, in addition to analyzing the *CCR5* +59029 (rs1799987) variant in the promoter region, which has been previously associated with a reduced risk of developing CCC in other reports (21–23), we also analyzed *CCR1* variants that were not previously explored in CD patients. The *CCR5* variant rs1799987 and the *CCR1* SNPs (rs3181077, rs1491961 and rs3136672) showed no significant association with CCC outcome. The increased frequency of asymptomatic CD patients showing the *CCR5* +59029G allele associated with low CCR5 expression raised the possibility that the decreased CCR5 expression in inflammatory cells infiltrating the heart tissue might protect against the development of CCC (22). In this vein, Brazilian CCC patients with ventricular dysfunction showed an increased frequency of the *CCR5* rs1799988CC genotype compared to those without dysfunction (46). However, *CCR5* +59029 (rs1799987) did not influence the left ventricular systolic dysfunction in patients with Chagas heart disease in another Brazilian group of patients (24). Therefore, together, these data are not conclusive regarding the influence of *CCR5* +59029 (rs1799987) on Chagas heart disease outcomes. In the present study, haplotype analysis of the *CCR1*–*CCR5* cluster showed no association with susceptibility to or severity of CCC. We further observed no correlation of CCL5 levels in serum with the *CCL5, CCR1*, and *CCR5* gene variants studied, supporting the conclusion that CCL5 serum levels may be controlled by other variants of these or other genes, or even that CCL5 serum levels may result from complex parasite-host interactions. Indeed, CCL5 expression by macrophages and cardiomyocytes is triggered by *T. cruzi* infection and upregulated by cytokines (52, 54).

Our finding supporting the association of *CCL5* rs2107538 variant T, which may upregulate CCL5 expression (49), with protection against the development of CCC, led us to question the possible biological pathways for the induction of protection by CCL5 in Chagas heart disease. To shed light on this point, we analyzed the participation of CCL5 and its receptors CCR1 and CCR5 in CCC using an experimental model of C57BL/6 mice infected with the Colombian strain, which reproduces critical aspects of Chagas heart disease (28, 37, 40, 41) and presents elevated levels of CCL5 in serum (55). Increased CCL5 levels were also detected in the cardiac tissue of chronically infected mice, corroborating the detection of enhanced CCL5 mRNA expression in this tissue (56). In models of acute and chronic Chagas heart disease in C3H/He mice, CCL5 signaling *via* CCR5/CCR1 has been proposed to play a critical role in cell migration and myocardial damage (16, 17). In oral *T. cruzi* infection, CCL5 is crucial for parasite growth control and mucosal immunity *via* B-cell activation and antibody production (53). However, the contribution of CC-chemokine receptors to nonbeneficial and/or protective CCL5-mediated interactions had not been completely elucidated (16, 17, 53). Increased frequencies of CCR5^+^ T cells and macrophages have been detected in the peripheral blood of CD patients compared with NI individuals (15, 51). Herein, we showed that in the spleen of chronically *T. cruzi*-infected mice, the increased frequencies of CCR5^+^ CD8^+^ T cells and CD14^+^ macrophages were associated with a decreased frequency of CCR1^+^ CD8^+^ T cells and CD14^+^ macrophages. In acute *T. cruzi* infection, the increased frequency of CCR5^+^ cells has been associated with TNF/TNFR1 signaling (57). Therefore, although not addressed in the present study, it is tempting to speculate that the local and/or systemic high TNF levels present in *T. cruzi*-infected hosts (14, 40) may, directly or indirectly, control CCR5 expression, favoring the CCL5–CCR5 axis, which may fuel the activation/differentiation of CCR5^+^TNF^+^ cells and contribute to a self-sustained scenario. Interestingly, increased frequencies of CCR5^+^TNF^+^ T cells were detected in patients with Chagas heart disease compared to patients with the indeterminate form (15), in a condition with a TNF-enriched systemic inflammatory milieu (14, 42). Particularly, considering the Th1 (TNF^+^, IFNγ^+^) nature of the inflammatory cells infiltrating the heart tissue (4, 58), the increased frequency of asymptomatic patients, compared to patients with the cardiopathic form, with the gene variant *CCR5* +59029G allele associated with low CCR5 expression supports the conclusion that reduced CCR5 expression may be beneficial for CD patients (22).

In our model of experimental CCC, a higher frequency of CCR5^+^ than that of CCR1^+^ CD8^+^ T cells was inflammatory (TNF^+^). More importantly, we provide evidence that in NI age-matched mice and in the CCC model in C57BL/6 mice, CD14^+^ macrophages are mostly segregated into CCR1^+^ and CCR5^+^ populations, where CCR1^+^ cells are predominantly IL-10^+^, whereas CCR5^+^ cells are TNF^+^. These data indicate that the condition is innate in these 5- to 6-month-old mice, and it is not drastically influenced by *T. cruzi* infection. There was, however, an important increase in the numbers of CD14^+^ macrophages in the spleens of *T. cruzi*-infected mice. These CD14^+^ macrophages were mainly Ly6C^neg^ F4/80^+^ (either CD11b^+^ or CD11b^neg^). Nevertheless, it remains to be clarified whether the *T. cruzi* parasite and/or intrinsic host molecules induce the activation and expansion of CD14^+^ macrophages. Recently, LPS has been shown to increase the frequencies of splenic CD14^+^ and bone marrow CD14^+^CD11b^+^ macrophages (59), indicating that pathogen molecules may selectively activate macrophage populations.

Studies of CCR1 expression in peripheral cells have not been conducted in chagasic patients; however, increased frequencies of CCR5^+^ CD8^+^ T cells and macrophages have been detected (15, 51). Although a similar analysis of simultaneous labeling for CC-chemokine receptors and cytokine expression is not available for CD patients, cardiopathic patients show a higher percentage of macrophages expressing TNF, whereas macrophages of patients with the indeterminate form are committed to IL-10 production after contact with parasites (60). In chronically *T. cruzi*-infected patients and mouse models, CD8^+^ T cells and macrophages are major components of the heart inflammatory infiltrate (4, 5, 56). During *T. cruzi* infection, the frequency of CCR5^+^ cells is precociously increased in the spleen (7 dpi) and later in the blood (14 dpi), while the heart tissue is colonized by inflammatory cells by 28 dpi (16, 61). Indeed, spleen-born CD8^+^ T cells and macrophages are involved in both heart tissue injury and repair (7, 8). Therefore, considering the potential functional antagonistic roles of CCR5^+^ and CCR1^+^ cells, we approached the participation of these cells in CCC by initially using CCR5-deficient mice. The reduction of myocardial injury, assessed by CK-MB activity levels in serum, in CCR5-deficient mice compared with CCR5-sufficient mice suggested that CCR5-mediated interactions were associated with CCC severity. Levels of CK-MB activity in serum have been associated with CCC severity in chronic *T. cruzi*-infected rhesus monkeys (38), CCC evolution in C57BL/6-infected mice (28) and CCC severity in rats (39) and mice (40, 41). Furthermore, CK-MB is a cardiac biomarker in serum that is associated with Chagas cardiomyopathy and is a predictor of mortality (62). Thus, the demonstration of reduced CK-MB activity in CCR5-deficient mice reinforces the notion that CCR5 mediates a deleterious effect in heart tissue. Interestingly, these *T. cruzi*-infected CCR5-deficient mice exhibited a reduced parasite load, which could also contribute to reduced myocardial damage. Hence, our data reinforce the conclusion that the CCR5-mediated response is not essential for *T. cruzi* growth control (16, 17, 53). However, these chronically *T. cruzi*-infected CCR5-deficient mice presented more intense myocarditis with macrophages, CD4^+^ and CD8^+^ cells. Thus, the beneficial role of CCR5 deficiency may reside in the depletion of CCR5^+^TNF^+^ cells rather than in reduced cell infiltration in heart tissue. Thus, beyond the intensity of heart inflammatory infiltrates, the quality of the immune response associated with myocarditis may determine the outcome of Chagas heart disease. This idea is supported by the previous demonstration that blockade of TNF by antibody administration to chronically infected mice reduced CCC severity (40). Indeed, the deleterious role of CCR5^+^TNF^+^ cells in CCC is also supported by the increased frequency of these cells in the peripheral blood of cardiopathic patients compared to that in patients presenting the indeterminate form of CD (15).

A critical point to be considered is the increase in CCL5 levels in the heart tissue of *T. cruzi*-infected CCR5-deficient mice, corroborating findings showing elevated CCL5 serum concentrations in orally infected *Ccr5*^−/−^ mice (53). In CCR5-deficient mice, CCL5 may signal *via* CCR1 and CCR3 and play a beneficial role in parasite control and/or have a direct protective action against CCC. This idea is partially supported by *in vitro* data showing that CCL5 activates human and mouse macrophages, leading to parasite control (18, 52). This initial observation has prompted further explorations to distinguish the participation of CCR1-mediated interactions in myocardial injury in *T. cruzi* infection. Thus, considering the similar expression of CCR1 in heart tissue and spleen of *Ccr5*^+/+^ and *Ccr5*^−/−^ mice, we abrogated CCR1 signaling in CCR5-deficient mice using the selective CCR5/CCR1 antagonist Met-RANTES (43). Abrogation of the protective role of CCR5 deficiency supports the conclusion that CCR1-mediated interactions are involved in protection against myocardial damage in *T. cruzi* infection. The functional role of CCR1^+^ cells as protectors of myocardial injury is not clear. Regarding the regulatory (IL-10) and inflammatory (TNF) antagonist profiles, our data support that CCR1^+^ CD8^+^ T cells are less inflammatory than CCR5^+^ CD8^+^ T cells. Moreover, in infected mice, splenic CCR1^+^ macrophages are mainly IL10^+^. In these functional property may reside the protective role of CCR1-mediated interaction in CCC. Met-RANTES-treated C57BL/6 mice and CCR5-deficient mice pretherapy showed reduced TNF concentrations in heart tissue. Furthermore, Met-RANTES administration to CCR5-deficient mice resulted in augmented TNF levels in the heart milieu, supporting the conclusion that CCR1^+^ cells may be important for the control of TNF expression in *T. cruzi* infection. Indeed, in a model of arthritis, CCR1 blockade or depletion enhanced systemic TNF production (63). In Colombian-infected mice, IL-10 limits *T. cruzi* burden and protects against fatal myocarditis (64). Importantly, IL-10 levels in serum are higher in asymptomatic patients, while TNF levels are higher in cardiopathic CD patients (65). Furthermore, TNF blockade in chronically infected mice favors IL-10 expression, increases the frequency of IL-10^+^ F4/80^+^ macrophages and reverses CCC (40), supporting our hypothesis that the protective role of CCR1^+^ cells may reside in this differential IL-10 profile. Therefore, our present proposal is that IL-10- and TNF-expressing cells, which express CCR1 and CCR5 on the cell surface, respectively, may respond differentially to CCL5 or other CC-chemokines and, therefore, antagonistically act on heart tissue during *T. cruzi* infection. Therefore, one putative way to favor a beneficial immune response would be to activate CCR1^+^ cells and CCL5 production in *T. cruzi*-infected individuals. To explore the viability of this idea, we stimulated peripheral blood cells of CD patients with *T. cruzi* antigens. Indeed, we observed a significant increase in the frequency of CCR1^+^, but not of CCR5^+^, CD8^+^ T cells, upregulated CCR1 expression and increased concentrations of CCL5 in supernatants. These effects were elevated in patients in group C (severe cardiopathy) compared with those in group A (non-cardiopathic). Theoretically, these newly antigen-experienced CD8^+^ T cells gain access to peripheral tissues and sites of inflammation, where they may exert their functions (66). Therefore, our data reinforce the possibility of a repositioning of the immune response toward a protective profile (in the present case, one associated with cell migration and functional properties), which can be achieved by therapeutic vaccine or antigen-driven stimulation, as a feasible strategy to improve the prognosis of CD patients.

Similar to most studies of gene polymorphisms in CD, our study also has some limitations related to sample size. Furthermore, the reduced number or lack of previous studies did not allow comparative analysis with our studied group of patients considering the same genetic markers. Our finding demonstrating a protective effect against CCC for the heterozygote CT and T allele *CCL5* rs2107538 variant should be validated in other populations. Additionally, some controversial data with respect to the literature may highlight a multigenic character of CCC, supporting the notion that genetic variants of multiple molecules may contribute small effects that together explain the complex expression of Chagas heart disease. Based on the perspective of our study using the experimental model of CCC, an important feature to be addressed in CD patients is the cell migration phenotype in association with functionality in circulating cells. More broadly, our data revealed that CCR1^+^ and CCR5^+^ CD14^+^ macrophages expressed segregated and antagonist cytokine profiles. Therefore, this antagonistic cell migration profile may become a target of further exploration to comprehend the participation of CD8^+^ T cells and resident or infiltrating spleen-borne macrophages in Chagas heart disease and other cardiac diseases (8). Nevertheless, the data presented herein open a new avenue for the study of cell effector functions and migration profiles, which are apparently susceptible to repositioning after a proper stimulus, with the aim of understanding the involvement of the immune response in Chagas heart disease and improving the prognosis of patients afflicted by this neglected disease.

## Ethics Statement

This study was carried out in accordance with the recommendations of the Ethics Committees of Fiocruz/RJ (541/09) and PROCAPE/UPE (80210/10). All subjects provided written informed consent in accordance with the Declaration of Helsinki. This study was carried out in strict accordance with the recommendations of the Guide for the Care and Use of Laboratory Animals of the Brazilian National Council of Animal Experimentation (http://www.cobea.org.br/) and Federal Law 11.794 (October 8, 2008). The Institutional Committee for Animal Ethics of Fiocruz (CEUA-Fiocruz-L004/09; LW-10/14) approved all experimental procedures used in the present study. All presented data were obtained from three independent experiments (Experiment Register Books #31 and 57, LBI/IOC-Fiocruz).

## Author Contributions

Conceived and designed the experiments: JL-V, AMB, LA-A, WO, MM, and AAS. Performed the experiments: JL-V, AMB, LA-A, AAS, IP, LR, VL, AM, AKAS, and AMB. Analyzed the data: AMB, LA-A, VL, and JL-V. Wrote the manuscript: AMB, LA-A, and JL-V. All authors read and revised the manuscript.

## Conflict of Interest Statement

The authors declare that the research was conducted in the absence of any commercial or financial relationships that could be construed as a potential conflict of interest. The handling Editor declared a shared affiliation, though no other collaboration, with one of the authors CCC.
