# Supplementary material for: Genetic Polymorphism at CCL5 Is Associated With Protection in Chagas’ Heart Disease: Antagonistic Participation of CCR1+ and CCR5+ Cells in Chronic Chagasic Cardiomyopathy
Source: Front Immunol. 2018 Apr 11;9:615. doi: 10.3389/fimmu.2018.00615 (PMC5904358; doi:10.3389/fimmu.2018.00615)
Supplement: Supplementary file 1 [file Data_Sheet_1.docx]

# SUPPLEMENTARY MATERIAL LEGENDS

**S1 Table.** TaqMan assays used for SNP genotyping.

**S2 Table.** Logistic regression results for haplotype analysis of single nucleotide polymorphisms located in *CCL2* and *CCL5* genes.

**S3 Table.** Logistic regression results for haplotype analysis of single nucleotide polymorphisms located in *CCR1* and *CCR5* genes.

**Figure S1.** CCL5 levels and association with *CCL5*, *CCR1* and *CCR5* genotypes in CD patients. Comparison of CCL5 levels in serum according to the presence of dominant and recessive allele carriers of *CCR5* +59029 rs1799987, *CCR1.CCR3* rs1491961, *CCR1* rs3136672, *CCR1.CCR3* rs3181077.

**Figure S2.** CCL2 levels and association with severity of disease, genotypes and LVEF in CD patients. **(A)** CCL2 levels in serum of individuals seronegative non-infected (NI) and seropositive for Chagas disease; ^*^, *P <* 0 05 (*t*-student test). **(B)** CCL2 concentrations in serum of CD patients grouped as A (asymptomatic, n = 20), B1 (mild CCC, n = 20) and C (severe CCC, n = 16). **(C)** Comparison of CCL2 serum levels according to the dominant (AA) and the mutant allele G carriers (Mann-Whitney test). **(D)** Correlation between the CCL2 serum concentrations and the left ventricular ejection fraction (LVEF) in patients; *p* = 0.5011, r^2^ = 0.0031 (Linear regression). **(E)** CCL2 concentrations in supernatants of cell cultures of CD patients grouped as A, B1 and C; ^***^, *P <* 0.001, antigen-stimulated *vs* non-stimulated cells of each group (ANOVA, Bonferroni posttest).

**Figure S3.** Phenotypic characterization of splenic CD8^+^ cells. C57BL/6 mice were infected with 100 trypomastigote forms of the Colombian *T. cruzi* strain and analyzed at 120 days postinfection. Splenic mononuclear cells of non-infected (NI) and *T. cruzi*-infected were obtained and stained for CD8, CD11c, TCR, CCR1 and CCR5. (**A**) Singlets (FSC-Lin x FSC-Area, R1) and dead-excluded cells (FSC-A x SSC-Lin, R2) were analyzed for expression of CD8 (histogram, R3), TCR, as marker for T-cells, and CD11c, a marker for dendritic cells in NI and *T. cruzi*-infected mice. Table summarizes the frequencies of CD8^+^TCR^+^ and CD8^+^CD11c^+^ cells. (**B**) After selection of singlets and exclusion of dead cells (R1/R2) and analysis of CD8 x TCR (dot plot, R4), CD8^+^ T-cells were analyzed for CCR1 and CCR5 expression (dot plot). Correlation between CCR1 and CCR5 expression on CD8^+^ T-cells of non-infected (black circles) and *T. cruzi*-infected (red circles) mice was determined by linear regression (r^2^ = 0.424). Data represent three independent experiments with 3 NI and 5-7 infected mice.

**Figure S4.** Cytokine profile in single-positive CCR1^+^ or CCR5^+^ cells among splenic CD8^+^ T-cells of *T. cruzi*-infected C57BL/6 mice. Mice were infected with 100 trypomastigote forms of the Colombian *T. cruzi* strain and analyzed at 120 days postinfection. Splenocytes were collected and stained for intracellular cytokines and cell surface markers (TNF, IL-10; CD8, CCR1, CCR5). Pie charts represent the fractions of CD8^+^ T-cells obtained from spleens of NI and *T. cruzi*-infected mice that carry each of the intracellular cytokine phenotype shown in the legend. Table summarizes the TNF/IL-10 ration in single-positive CCR1^+^ or CCR5^+^ CD8^+^ T-cells. Data represent two independent experiments with 3 NI and 5-7 infected mice. ^*^, *P <* 0.05 NI *vs* *T. cruzi*; ^#^ *P <* 0.05 CCR1^+^ *vs* CCR5^+^ cells (*t*-student test).

**Figure S5.** Representative data of phenotypic characterization of splenic CD14^+^ cells. C57BL/6 mice were infected with 100 trypomastigote forms of the Colombian *T. cruzi* strain and analyzed at 120 days postinfection. (**A**) Splenic mononuclear cells of non-infected (NI) and *T. cruzi*-infected were obtained and stained for CD14, Ly6c, CD11c: singlets (FSC-Lin x FSC-Area, R1) and dead-excluded cells (FSC-A x SSC-Lin, R2), were analyzed for CD14 x Ly6C dot plot or CD14 x CD11c dot plot. (**B**) Splenic cells were stained for CD14, CD45R, F4/80 and CD11b: singlets (FSC-Lin x FSC-Area, R1) and dead-excluded cells (FSC-A x SSC-Lin, R2), were analyzed for CD14 x CD45R dot plot; CD14^+^CD45R^+^ gated cells (R4) were analyzed for CD11b x F4/80 dot plot. (**C**) Relative spleen weight (spleen weight in mg / body weight in g), cellularity and numbers of CD14^+^ macrophages were determined. Data represent two independent experiments with 3 NI and 5-7 infected mice. ^*^, *P <* 0.05, ^**^, *P <* 0.01 *T. cruzi*-infected mice *vs* NI (*t*-student test).

**Figure S6.** Representative analysis of cytokine profiles in single-positive CCR1^+^ or CCR5^+^ cells among splenic CD14^+^ macrophages of *T. cruzi*-infected C57BL/6 mice. C57BL/6 mice were infected with 100 trypomastigote forms of the Colombian *T. cruzi* strain and analyzed at 120 days postinfection. Splenic mononuclear cells of non-infected (NI) and *T. cruzi*-infected were obtained and stained for intracellular cytokines and cell surface markers (CD14, CCR1, CCR5, TNF, IL-10). (**A**) After selection of singlets (FSC-Lin x FSC-Area, R1) and dead-cell exclusion (FSC-A x SSC-Lin, R2), CD14^+^ cells (R3) were analyzed for IL-10 x TNF dot plot. (**B**) CD14^+^ cells (R3) were analyzed for CCR1 x CCR5 dot plot and single-positive CCR1^+^ (R4) or CCR5^+^ (R7) cells were analyzed for IL-10 x TNF dot plots. (**C**) Pie charts represent the fractions of CD14^+^ macrophages obtained from spleens of NI and *T. cruzi*-infected mice that carry each of the intracellular cytokine phenotype shown in the legend. Data represent two independent experiments with 3 NI and 5-7 infected mice.

**Figure S7.** Effects of CCR5-deficiency in parasitemia, parasite load and inflammation in the heart tissue of *T. cruzi*-infected mice. Mice were infected with 1000 trypomastigote forms of the Colombian *T. cruzi* strain and analyzed at 70 days postinfection. Parasitemia was determined in peripheral blood and parasite load and inflammation in the heart tissue sections of *T. cruzi*-infected mice were analyzed by immunohistochemical assay. Data represent two independent experiments with 4-6 mice per group. ^*^, *P <* 0.05, ^***^, *P <* 0.001, *ccr5*^-/-^ *vs* *ccr5*^+/+^ *T. cruzi*-infected mice (*t*-student test).

**Figure S8.** Effects of CCR5-deficiency in myocardial injury and CCR1 expression in heart tissue and spleen. Mice were infected with 100 trypomastigote forms of the Colombian *T. cruzi* strain. **(A)** Kinetic of CK-MB activity in serum at 30, 60 and 120 days postinfection. **(B)** CCR1 expression in heart tissue and spleen sections of *T. cruzi*-infected mice pre-therapy (120 dpi) were analyzed by immunohistochemical assay. Data represent two independent experiments with 4-6 mice per group. ^*^, *P <* 0.05, *T. cruzi*-infected mice *vs* NI; ^#^, *P <* 0.05, *ccr5*^-/-^ *vs* *ccr5*^+/+^ *T. cruzi*-infected mice (ANOVA, Bonferroni posttest).

**Figure S9.** Effects of Met-RANTES treatment in TNF expression is the heart tissue of *T. cruzi*-infected mice. Mice were infected with 100 trypomastigote forms of the Colombian *T. cruzi* strain and treated with Met-RANTES (Met-R, 10 µg/mice) from 120-150 days postinfection. Hearts were collected at 150 dpi (post-therapy) for evaluation of expression of TNF by immunohistochemical assay. Data represent two independent experiments with 3-5 NI and 7-10 infected mice. ^#^, *P <* 0.05 and ^###^, *P <* 0.001, pre-treated *vs* Met-R-treated; ^&&&^, *P <* 0.001, *ccr5*^-/-^ *vs* *ccr5*^+/+^ *T. cruzi*-infected mice pre-treatment (120 dpi); ^ΨΨ^, *P <* 0.01, *ccr5*^-/-^ *vs* *ccr5*^+/+^ *T. cruzi*-infected mice post-treatment (150 dpi) with Met-R (ANOVA, Bonferroni posttest).

**S1 Table.** TaqMan assays^a^ used for SNP genotyping.

| **Gene** | **Location**^b^ | **SNP** | **dbSNP** | **TaqMan Assay ID** |
| --- | --- | --- | --- | --- |
| ***CCR1*** | chr 3: 46209161 | x | rs3181077 | C___1198056_10 |
| ***CCR1*** | chr 3: 46208857 | x | rs1491961 | C__11539883_30 |
| ***CCR1*** | chr 3: 46201294 | x | rs3136672 | C___1198067_10 |
| ***CCR5*** | chr 3: 46370444 | +59029 | rs1799987 | Custom designed assay |
| ***CCL2*** | chr 17: 34252769 | -2518 | rs1024611 | C___2590362_10 |
| ***CCL5*** | chr 17: 35880776 | -403 | rs2107538 | C__15874407_10 |
| ***CCL5*** | chr 17: 35880401 | -28 | rs2280788 | C__15874396_20 |

^a^ ThermoFisher Scientific, USA.

^b^ Location is based on GRCh38 assembly build.

**S2 Table.** Logistic regression results for haplotype analysis of single nucleotide polymorphisms located in *CCL2* and *CCL5* genes. Haplotype frequencies are shown by group.

| **rs1024611/rs2107538/rs2280788** | | | | | | | | | **A versus B1** | | | **A versus C** | | | | **B1 versus C** | | | | |
| --- | --- | --- | --- | --- | --- | --- | --- | --- | --- | --- | --- | --- | --- | --- | --- | --- | --- | --- | --- | --- |
|  | **Stage A**  **N = 110** | | **Stage B1**  **N = 163** | | **Stage C**  **N = 133** | | **OR**^a^  **[95% CI]** | | | ***P*-value** | | | **OR**^a^  **[95% CI]** | ***P*-value** | | | **OR**^a^  **[95% CI]** | | ***P*-value** | |
| A/C/G | | 0.46 | | 0.51 | | 0.54 | | - Reference - | | | | | | | | | | | | |
| A/T/C | | 0.01 | | 0.02 | | 0.02 | | 1.1 [0.1-10.4] | | | 0.91 | | 0.9 [0.1-11.3] | | 0.95 | | | 0.6 [0.1-3.7] | | 0.60 |
| A/T/G | | 0.21 | | 0.14 | | 0.15 | | 0.7 [0.3-1.3] | | | 0.23 | | 0.6 [0.3-1.2] | | 0.19 | | | 1.0 [0.6-1.9] | | 0.93 |
| G/C/G | | 0.22 | | 0.25 | | 0.23 | | 1.4 [0.8-2.7] | | | 0.28 | | 1.1 [0.6-1.9] | | 0.85 | | | 0.8 [0.5-1.4] | | 0.54 |
| G/T/G | | 0.1 | | 0.09 | | 0.07 | | 0.7 [0.3-1.3] | | | 0.26 | | 0.5 [0.2-1.2] | | 0.11 | | | 0.7 [0.3-1.6] | | 0.43 |

^a^ Odds ratio (OR) values shown are corrected for gender and ethnicity.

Abbreviations: CI = confidence interval.

**S3 Table.** Logistic regression results for haplotype analysis of single nucleotide polymorphisms located in *CCR1* and *CCR5* genes. Haplotype frequencies are shown by group.

| **rs3136672/rs1799987/rs1491961/rs3181077** | | | | **A versus B1** | | **A versus C** | | **B1 versus C** | |
| --- | --- | --- | --- | --- | --- | --- | --- | --- | --- |
|  | **Stage A**  **N = 110** | **Stage B1**  **N = 163** | **Stage C**  **N = 133** | **OR**^a^  **[95% CI]** | ***P*-value** | **OR**^a^  **[95% CI]** | ***P*-value** | **OR**^a^  **[95% CI]** | ***P*-value** |
| G/A/C/T | 0.36 | 0.30 | 0.32 | - Reference - | | | | | |
| A/A/C/T | 0.07 | 0.09 | 0.09 | 2.0 [0.8-5.5] | 0.16 | 1.9 [0.6-5.7] | 0.27 | 0.8 [0.4-1.9] | 0.67 |
| A/G/C/T | 0.08 | 0.09 | 0.13 | 1.6 [0.7-3.6] | 0.25 | 1.7 [0.8-3.8] | 0.19 | 1.3 [0.6-2.6] | 0.47 |
| G/A/T/C | 0.10 | 0.12 | 0.14 | 1.3 [0.6-2.8] | 0.46 | 1.5 [0.7-3.4] | 0.30 | 1.1 [0.6-2.3] | 0.74 |
| G/G/C/T | 0.36 | 0.37 | 0.28 | 1.2 [0.7-2.2] | 0.46 | 1.0 [0.6-1.7] | 0.94 | 0.8 [0.5-1.3] | 0.35 |
| G/G/T/C | 0.03 | 0.03 | 0.04 | 1.3 [0.3-6.0] | 0.76 | 2.0 [0.5-8.4] | 0.33 | 2.1 [0.6-7.4] | 0.27 |

^a^ Odds ratio (OR) values shown are corrected for gender and ethnicity.

Abbreviations: CI = confidence interval.
