# Supplementary figures and images for: Genetic Polymorphism at CCL5 Is Associated With Protection in Chagas’ Heart Disease: Antagonistic Participation of CCR1+ and CCR5+ Cells in Chronic Chagasic Cardiomyopathy
Source: Front Immunol. 2018 Apr 11;9:615. doi: 10.3389/fimmu.2018.00615 (PMC5904358; doi:10.3389/fimmu.2018.00615)

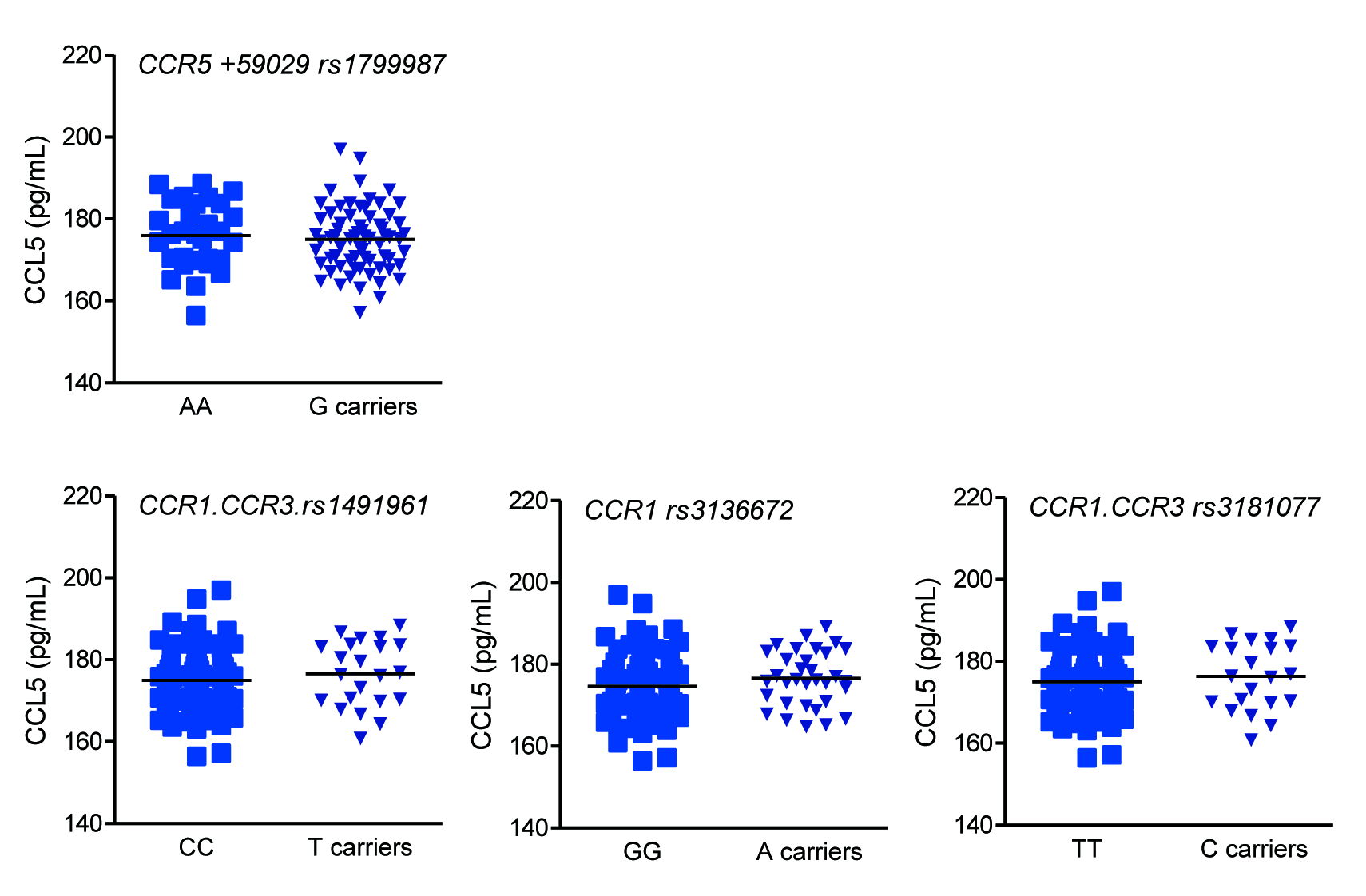

Supplement: Supplementary file 2 [file Image_1.tif]

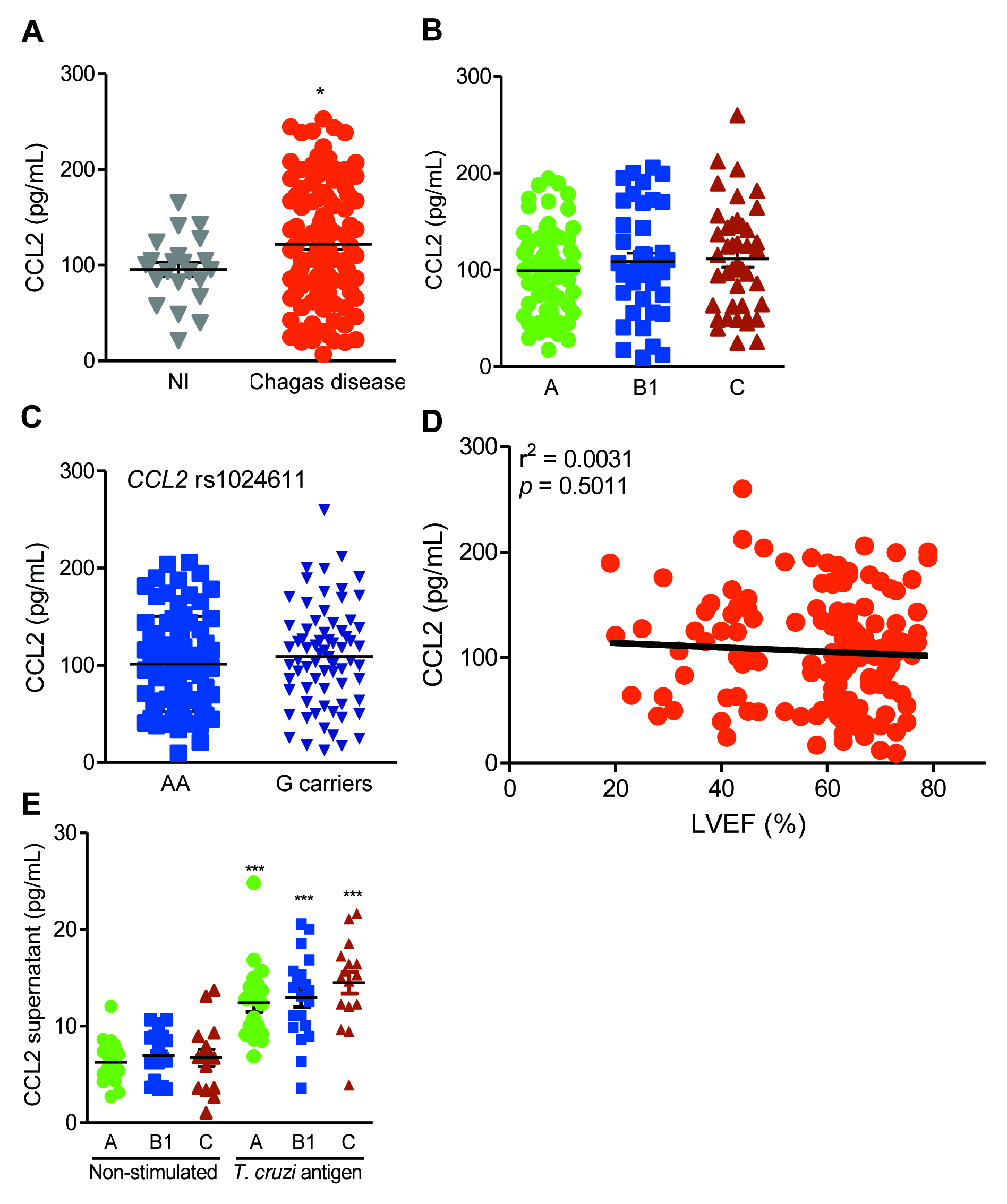

Supplement: Supplementary file 3 [file Image_2.tif]

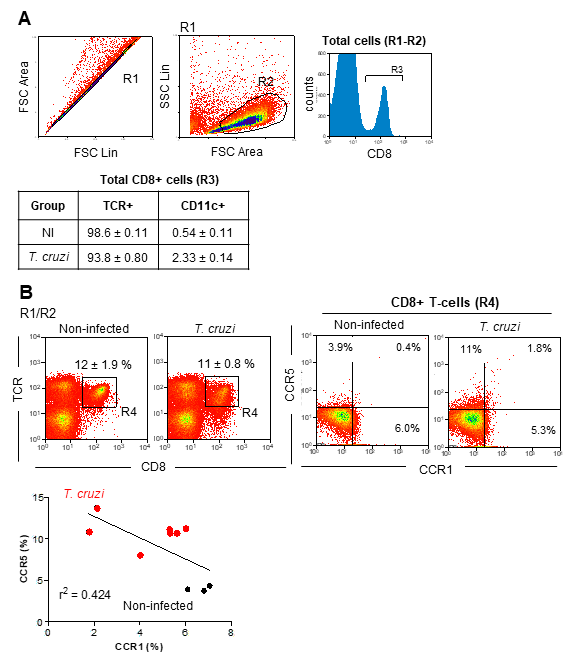

Supplement: Supplementary file 4 [file Image_3.tif]

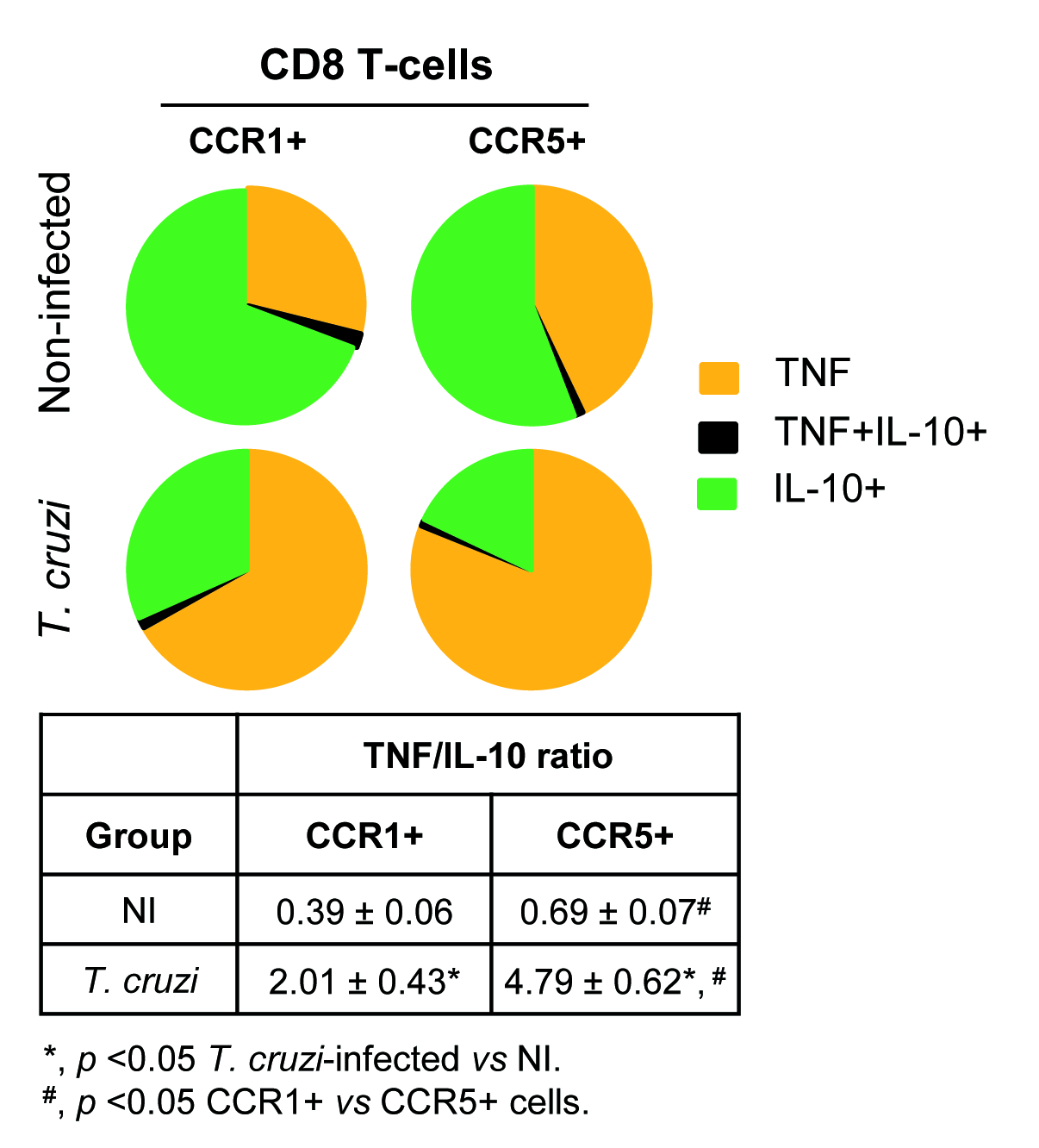

Supplement: Supplementary file 5 [file Image_4.tif]

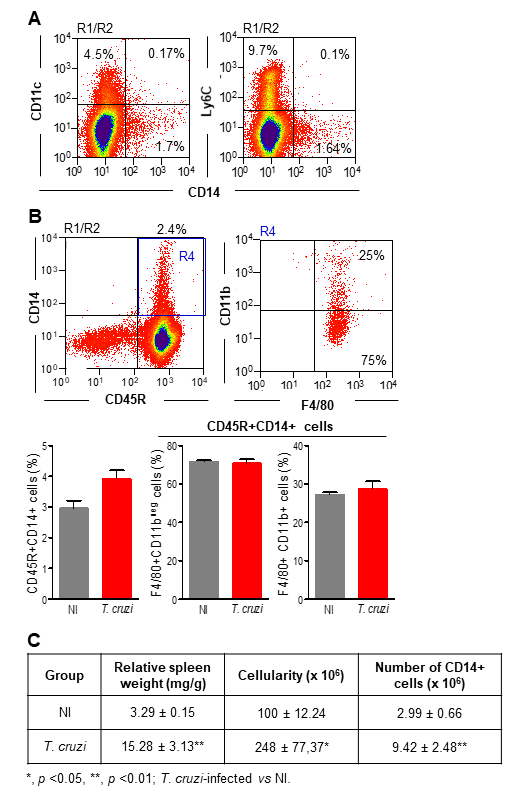

Supplement: Supplementary file 6 [file Image_5.tif]

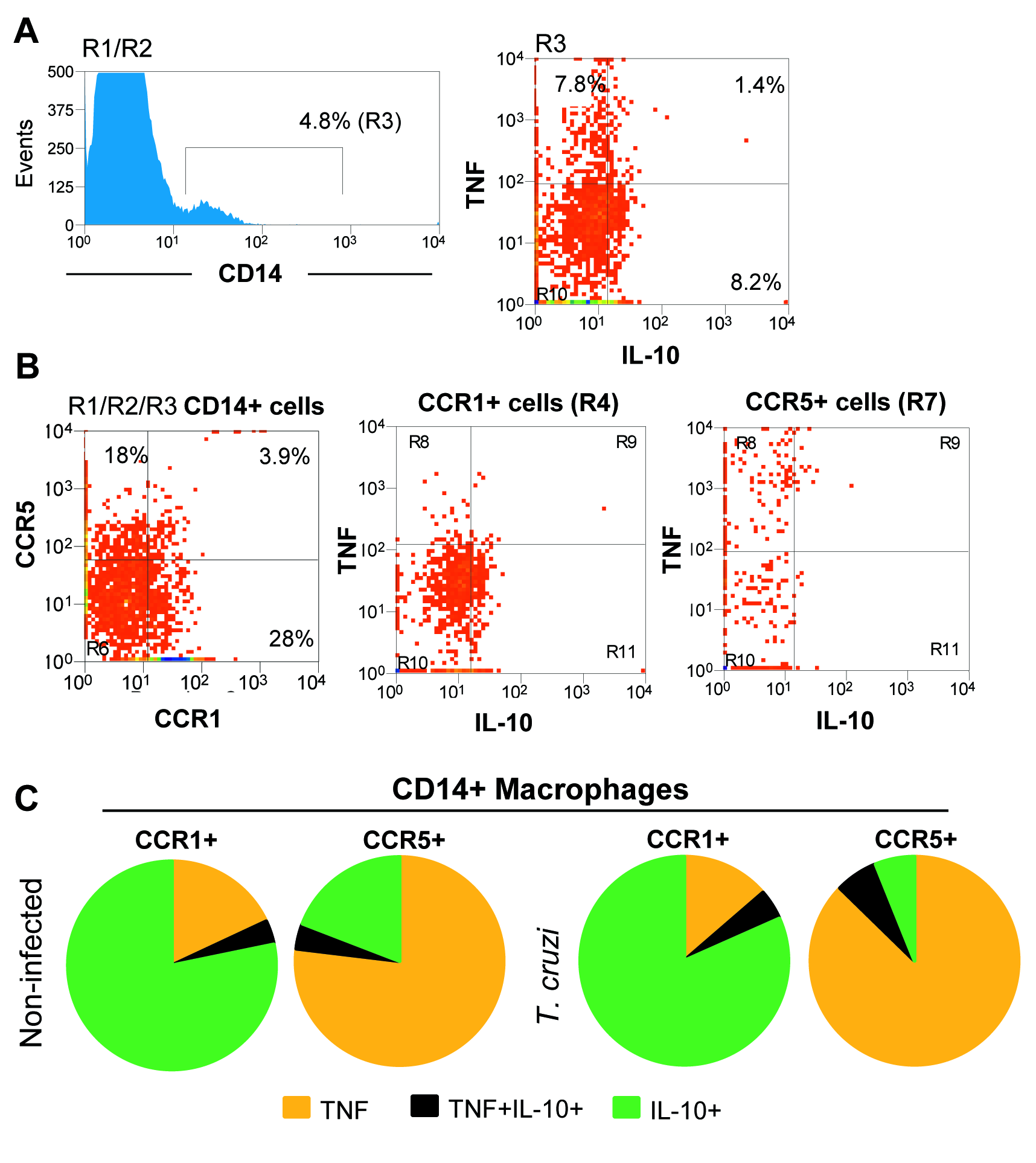

Supplement: Supplementary file 7 [file Image_6.tif]

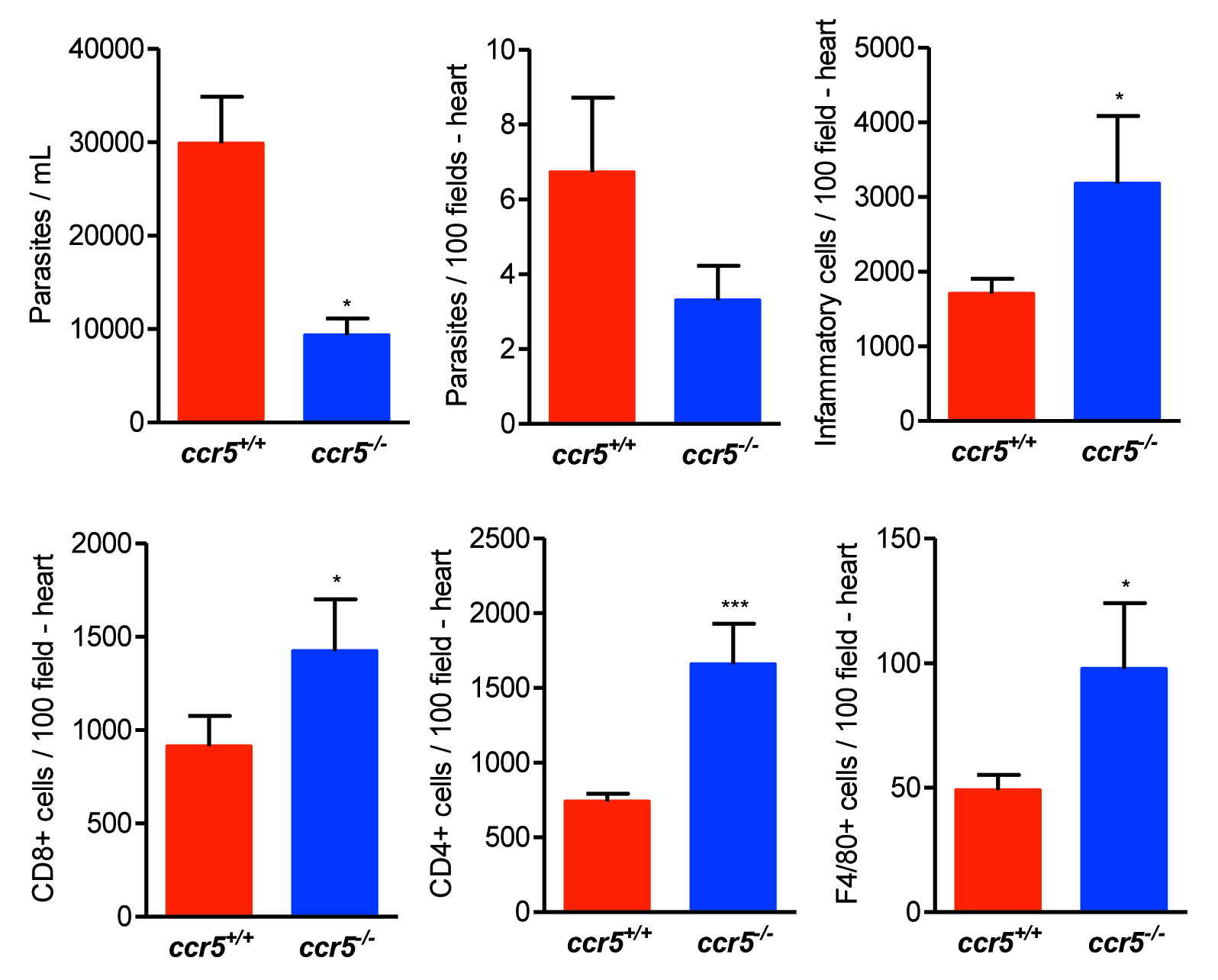

Supplement: Supplementary file 8 [file Image_7.tif]

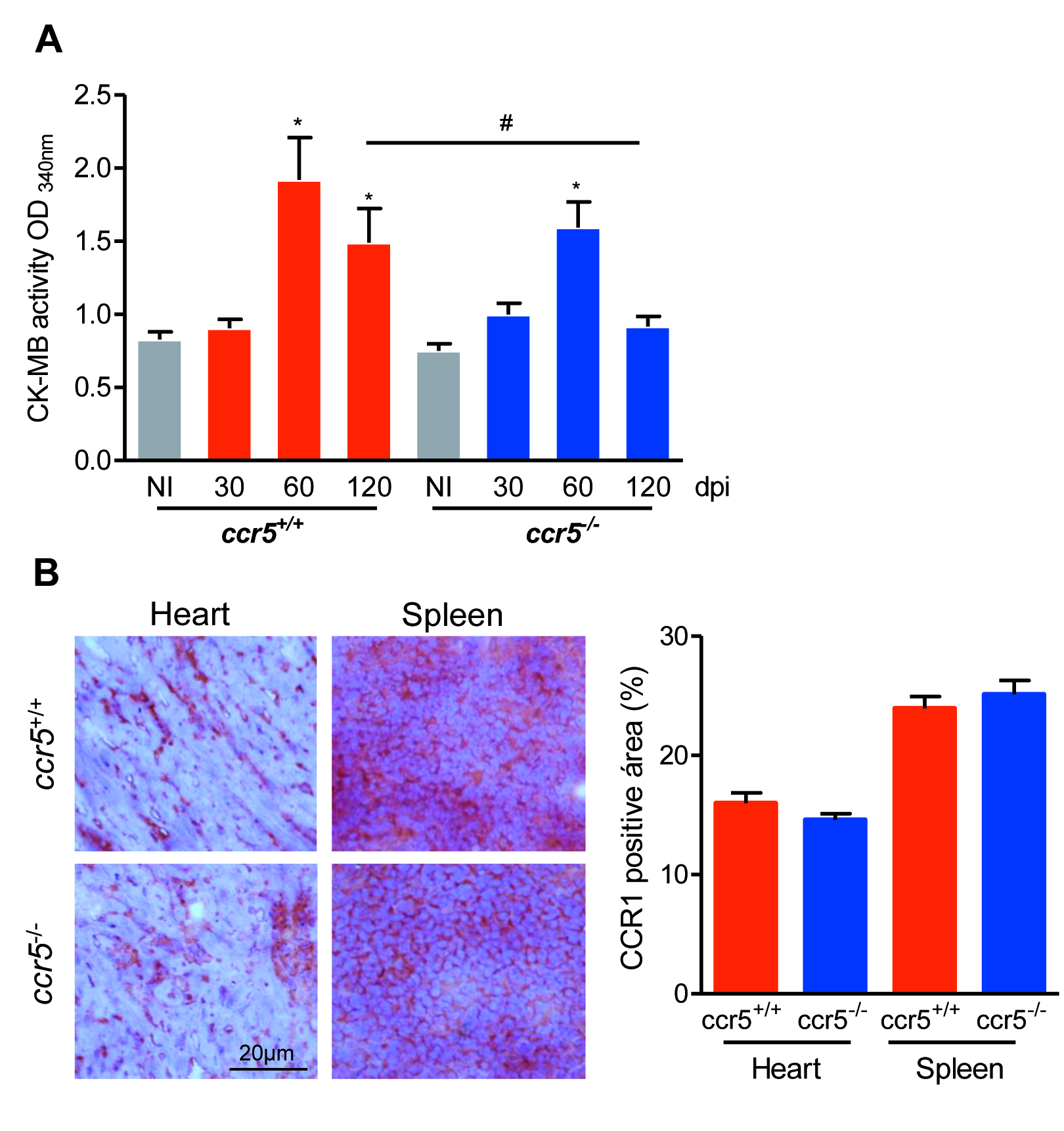

Supplement: Supplementary file 9 [file Image_8.tif]

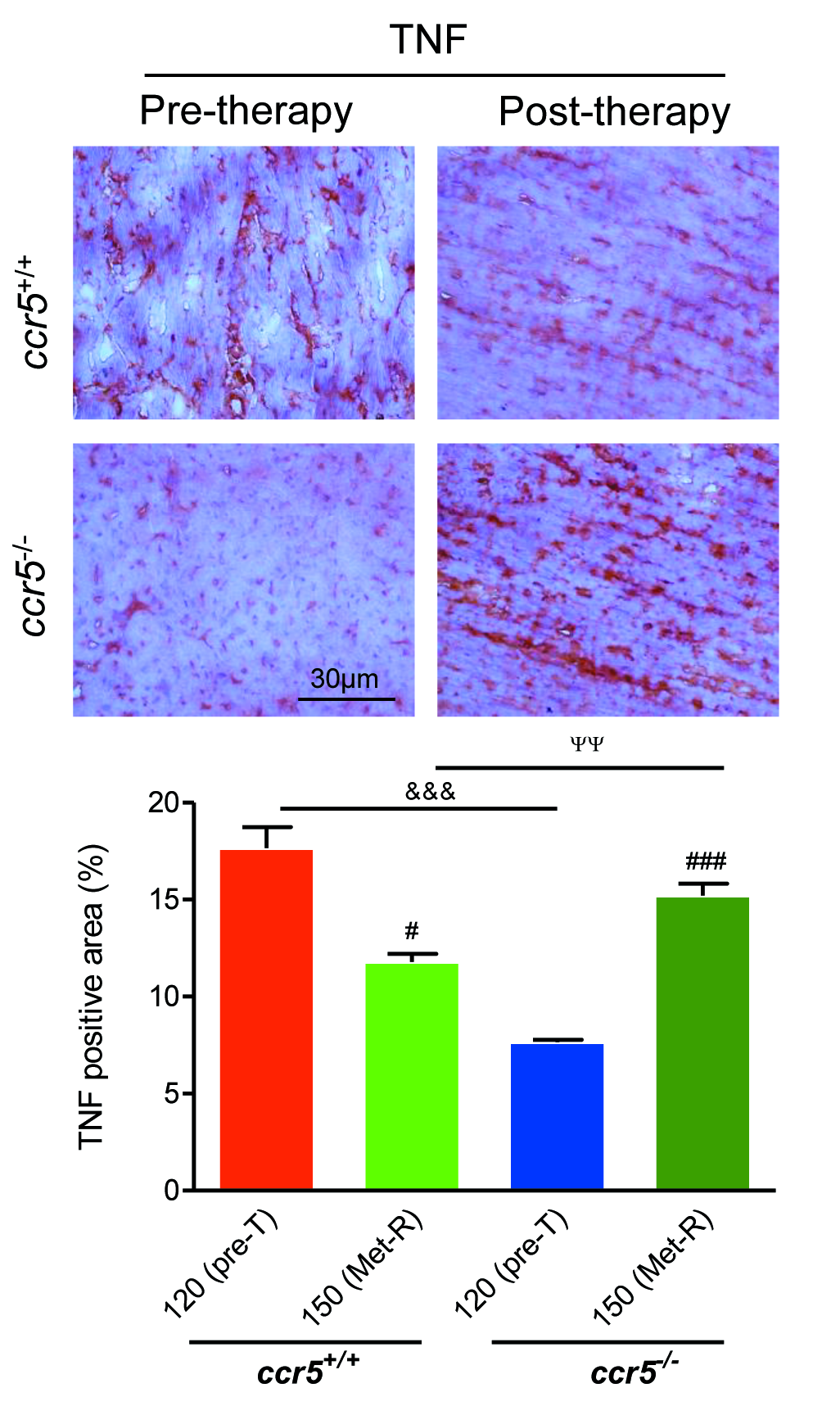

Supplement: Supplementary file 10 [file Image_9.tif]
